# Online Community Support for Stroke Survivors and Caregivers: Scoping Review

**DOI:** 10.2196/71190

**Published:** 2026-04-29

**Authors:** Putu Wuri Handayani, Kamila Alifia, Juliana Sutanto, Erlin Erlina, Sharyn Graham Davies, Arthur HP Mawuntu, Muhammad Isman Jusuf, Narelle Warren

**Affiliations:** 1 Faculty of Computer Science, Universitas Indonesia, Depok, Indonesia; 2 Department of Human Centred Computing, Faculty of Information Technology, Monash University, Clayton, Victoria, VIC 3800, Australia, +61 399026000; 3 Department of Health Behaviour, Environment and Social Medicine, Faculty of Medicine, Public Health and Nursing, Universitas Gadjah Mada, Yogyakarta, Indonesia; 4 School of Languages, Literature, Cultures and Linguistics, Faculty of Arts, Monash Univertisty, Melbourne, Victoria, Australia; 5 Department of Neurology, Faculty of Medicine, Universitas Sam Ratulangi, Manado, Indonesia; 6 Department of Neurology, Faculty of Medicine, Universitas Negeri Gorontalo, Gorontalo, Indonesia; 7 School of Social Sciences, Faculty of Arts, Monash University, Wellington Rd, Melbourne, Victoria, VIC 3800, Australia

**Keywords:** scoping review, stroke, online community, online community assistance, digital technology

## Abstract

**Background:**

Previous studies found that online communities are critical in supporting stroke survivors and caregivers for stroke recovery. However, it is unclear how such online communities are designed, or could be designed.

**Objective:**

This review aims to identify the key design elements of an online community to support stroke survivors and caregivers, that is, the actors, types of community support, and supporting technologies.

**Methods:**

We used the Preferred Reporting Items for Systematic Reviews and Meta-Analyses Extension for Scoping Reviews (PRISMA-ScR) guidelines. We included journal articles related to online community support for stroke. Editorials, registers, opinion pieces, letters, and conference papers were excluded. Online databases PubMed/MEDLINE, Scopus, Web of Science, ScienceDirect, and ProQuest were searched for articles published from January 2015 to June 2025. Articles were screened based on the title, abstract, and full text using Covidence software. After screening and full-text review, we read the selected articles in detail to analyze and synthesize information on key actors, types of support, and technologies used to support stroke survivors and caregivers.

**Results:**

A total of 77 articles were included. These articles discussed digital support technologies (52 articles), community functions and roles (18 articles), online stroke community systems (6 articles), and the stroke ecosystem (1 article). Our review found that the online community of support for stroke survivors and caregivers includes the caregivers themselves (46 articles), health workers (24 articles), and the local community/society (14 articles). Online communities mainly provide informational support, including giving advice (28 articles) and tangible aids (29 articles), followed by social support to create a sense of belonging (26 articles). Technologies discussed included mobile health (25 articles), web-based systems (12 articles), virtual/augmented reality (8 articles), sensors/wearable technology (8 articles), video-guided exercise apps (4 articles), and telehealth/telerehabilitation/teleconsultation/telestroke (4 articles). Only one examined how cultural differences influence technology.

**Conclusions:**

Although technologies are essential in online communities of support for stroke survivors and caregivers, this review shows a lack of studies that analyze the use and role of technologies in such online communities. This could be because the key actors of the online communities are the caregivers, who mainly seek social support and therefore do not require sophisticated technology. Nevertheless, technologies such as telerehabilitation and video-guided exercise apps could be important for other actors, including the local community and health workers, to enable them to support stroke survivors and their caregivers.

## Introduction

### Background

Globally, stroke is the second leading cause of death and third leading cause of disability [1]. Caused by impaired perfusion through the blood vessels to the brain, stroke requires high quality, fast, and precise management to prevent and avoid disability and death [1-3]. Furthermore, stroke causes chronic effects, including mobility problems, cognitive impairment, and depression, that can become a burden on health care systems [4]. Although the time to discharge varies considerably between and within countries, most stroke survivors are discharged home with differing degrees of residual physical impairments [5], leading to an increased reliance on their caregivers. Caregivers are often a spouse or partner, family member, friend, or significant other who provides physical, practical, transportation, and emotional help [6-8].

Regular rehabilitation therapy has been demonstrated to improve physical and cognitive capacity and quality of life for stroke survivors [9-11]. Post discharge from the acute care setting, stroke survivors should ideally undergo regular reviews of their health and social status by their primary care service provider, typically at 6 weeks, 6 months, and then annually [12]. However, the extent to which this can be realized varies for multiple reasons. Challenges encountered by stroke survivors in seeking to access appropriate health facilities for treatment or rehabilitation include: complex pathways to care, limited physical mobility, travel distances, limited transportation options, long waiting times, and unaffordable out-of-pocket expenses [10,13].

Technologies such as teleconsultation and telerehabilitation offer stroke survivors and their caregivers’ ways to address these barriers [14], offering ways to receive treatment and education materials from doctors or therapists remotely. However, effective use of such technologies requires digital literacy and access: stroke survivors and caregivers who are not technology savvy may avoid using the technologies and instead seek help from others in their community [15,16], if at all. Digital literacy and access to technologies are especially important for stroke survivors living in rural or regional areas in low- and middle-income countries (LMICs) [17].

**Figure 1.**
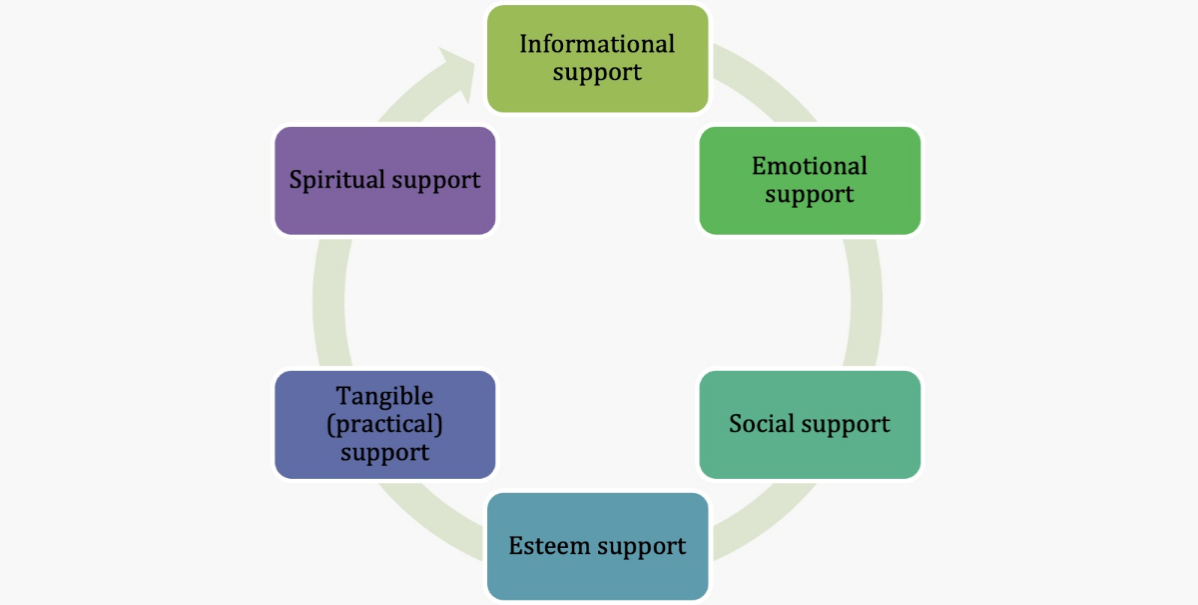
Multidimensional support needs of community-based stroke survivors and their caregivers.

Community—including groups of families, individuals, professionals, organizations, or other types of networks and social circles—can play an essential role in stroke recovery and rehabilitation [10,18-22]. Wright et al [23] found that long-term stroke survivors’ engagement with the community was key to addressing their multidimensional support needs (Figure 1). Moreover, Intamas et al [21] suggested that developing a stroke care model by involving the community could improve the quality of life of stroke survivors and caregivers.

Informational support includes education provision (including on stroke symptoms and improved stroke identification) [24,25], sharing resources for raising awareness, and linking people to services and treatment options [26]. Emotional support addresses psychological concerns about the impact of stroke, recovery progress, and addressing worries about the future (including risk of future stroke) [27,28], including reassurance, encouragement, affirmations, and messages of care, concern, and empathy [29,30]. Social or network support seeks to address loneliness or social isolation that can follow a stroke by promoting a sense of belonging and connection. Social support may also include support to engage in social activities (eg, clubs or events), physical activity or exercise programs [29-31], while esteem support focuses on a person’s intrinsic value and abilities to reinforce their personhood and social identity [32,33]. These aspects are complemented by sensitive tangible (practical) support, which focuses on assistance in activities of daily life [34]. Spiritual support may also play an important role at the community level; for example, Smith et al [8] found that an online community could give “prayer support.” This prayer or spiritual support appeals to people’s religious or spiritual beliefs to create a sense of connection to cope with their illness [8,35-38].

Research in all contexts has highlighted the significance of community-based interventions in facilitating poststroke recovery and reintegration [16,39]. Health professionals rely on community-based initiatives to assist and monitor stroke survivors [8], share tasks associated with stroke care [17], and promote stroke prevention [40]. Community-based initiatives are especially important in LMIC, where health workforce and infrastructure issues limit the formal provision of stroke care, leaving the bulk of stroke survivorship care and management to those located at the household or community level [17,41,42].

### Objectives

Despite the importance of community-based support for stroke survivors and their caregivers, there remain challenges to its delivery in many settings. Online support strategies offer much promise in addressing stroke survivors’ and caregivers’ needs [43,44], but how an online health community of support should be designed and what components it should include remains unclear. The aim of this scoping review is therefore to (1) identify the key actors for an online health community, (2) define the types of support to be incorporated in an online platform, and (3) determine the technological aspects of an online health community of support. These three key elements (who, what, and how) are the pillars in designing an online community of support for stroke survivors and caregivers.

## Methods

### Study Design

This scoping review used the Preferred Reporting Items for Systematic Reviews and Meta-Analyses extension for Scoping Reviews (PRISMA-ScR) to guide its planning, designing, and reporting [45]. The PRISMA-ScR approach was adopted in order to provide an understanding of the relevant core concepts and key items, as well as to guide the synthesis of the search results, and the Preferred Reporting Items for Systematic Reviews and Meta-Analyses (PRISMA) [46] guidelines were followed throughout (as detailed in Checklist 1).

### Eligibility Criteria

We used the population, concept, and context framework to define our eligibility criteria, as shown in Textbox 1. Articles that did not focus on online community support for stroke were excluded from this study. Primary studies and review articles were included. Review articles were important in our review for an overview of the prevailing concept of online community for stroke support and the technology needed for an online health support community. We excluded editorials, registers, letters, and conference papers. Only English-language articles were included, as English is the dominant language of scientific communication, and in order to ensure feasibility, consistency, and accuracy in data extraction. We searched for articles published between January 2015 and June 2025; 2015 marks an increased use of technologies for managing stroke.

Textbox 1. **Eligibility criteria.**

**Inclusion criteria**

Population: Actors that engage in an online stroke community

Concept: An online community providing stroke support, with a focus on technology-enabled health solutions

Context: Health study or health technologies on stroke in the online community

Study Design: Primary study with any design and review study

Study Type: Published in a peer-reviewed journal from January 2015 to June 2025

Language: English

Access: Full-text available

**Exclusion criteria**

Population: Actors not engaged in an online stroke community

Concept: Not stroke-related studies and not focused on an online community of support for stroke survivors

Context: Not health study or health technologies on stroke in the online community

Study Design: Editorials, registers, letters, and conference papers

Study Type: Conference papers, dissertations, books

Language: Language other than English

Access: Full-text not available

### Information Sources and Search Strategy

Online databases PubMed/MEDLINE, Scopus, Web of Science, ScienceDirect, and ProQuest were searched due to their comprehensive coverage of health research journals. Based on the study aims and eligibility criteria, the following search (keywords) terms were used for all database searches: online health community, online health support community, health support community, health ecosystem, technology, and stroke.

Multimedia Appendix 1 describes the step-by-step search strategy for each online database and outlines the slightly different search mechanisms used. For example, ScienceDirect does not permit the use of wildcards (the * extension), and we used the “tiab” (title and abstract) topic search mechanism for PubMed and ScienceDirect, while ProQuest uses the “noft” (anywhere except full text) tag for topic searches. We used the “OR” Boolean operator to create search terms among the free-text keywords related to stroke, online community, and technology, as well as medical subject heading terms. Individual search terms (Multimedia Appendix 1) were then combined using the “AND” Boolean operator. We used medical subject heading terms related to the concept of stroke (ie, stroke, poststroke, cerebrovascular accident, and stroke survivor), online community (ie, online community, virtual community, support group, peer support, social support, online forum, self-help groups, social environment, caregiver), and technology (ie, eHealth, mobile health [mHealth], web-based, digital, virtual, online, software, internet-based intervention, virtual reality [VR], apps).

The difference between a community and an online community lies only in the use of online channels or digital technology used in carrying out activities. Thus, online community support includes all kinds of support via online channels or digital technology.

Search strings were applied one by one in the selected online databases. PWH and NW searched PubMed/MEDLINE and Web of Science as well as ProQuest, and KAI searched Scopus. All articles were uploaded into Covidence software by PWH, KAI, and NW. PWH, KAI, JS, NW, SD, AM, and MIJ screened articles by using the Covidence software, which was used for title and abstract screening and for full-text review. Covidence allowed identification of duplicate articles [47]. Title and abstract screening were done by PWH and KAI and verified by JS and NW. Screening for full-text articles was then undertaken by all authors independently, and we discussed the results for final consensus. Where any conflicts existed in decision-making, JS and NW made a final determination through discussion.

### Data Extraction and Synthesis

A data chart was created using Microsoft Excel to identify which variables to extract. Two reviewers (PWH and KAI) independently and iteratively charted the data, wrote, and discussed the results. Additional sources, such as supplementary material, were analyzed to support the data charting.

Data were extracted based on the aims and objectives of each article, as well as data that fit with the scoping review objectives. Additional data on article demographics were extracted to capture: (1) the article’s central topic, (2) country or countries where the study was conducted, (3) the methodology used, (4) the actors in the community (if relevant), (5) the type and nature of community support for stroke survivors and caregivers, and (6) the supporting technology developed or used. The full data chart is provided in Multimedia Appendix 2 [3,4,7-10,12,14,15,23,39,48-113], and the cited journals are summarized in Multimedia Appendix 3. Descriptive analyses were conducted to identify key research themes before grouping the articles based on these defined themes.

## Results

### Study Selection

#### Overview

**Figure 2.**
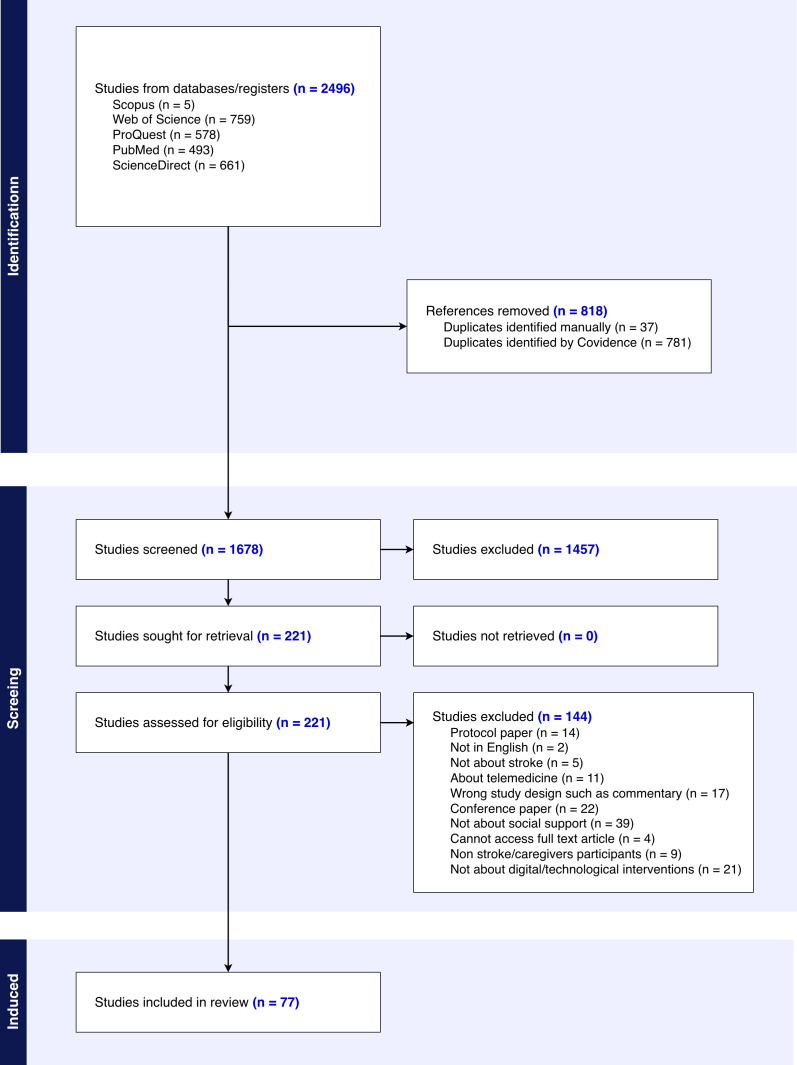
PRISMA (Preferred Reporting Items for Systematic Reviews and Meta-Analyses) flow diagram.

In this review, 2496 articles were imported from the literature searches into Covidence for screening. We removed 818 duplicates; 1457 studies were deemed irrelevant, as they did not focus on stroke. The remaining 221 articles were assessed for full-text eligibility. A total of 144 articles were excluded because they did not solely focus on stroke, were protocol papers, or the topics were unrelated to our study objectives. Figure 2 shows the PRISMA flow diagram, where a total of 77 articles were selected.

#### Article Demographic Summaries: Topic, Location, and Methodologies Used

Data on the main themes were extracted. The 77 articles addressed a range of topics, including: (1) digital technologies used to support people with stroke (52 articles), (2) the function and roles of community in stroke recovery (18 articles), (3) online stroke community systems (6 articles), and (4) ecosystems of stroke (1 article), that involve relevant actors, such as online communities, organizations, and implementation partners to manage, implement, and disseminate tangible actions to reduce stroke burden. Table 1 identifies the distribution of relevant articles (by reference list number) by topic. Each article has one topic group.

**Table 1.** Distribution of articles across topic areas.

Topic	Reference list number	Number of papers (%)
Digital support technologies	Thompson et al [4], Scrivener et al [48], Camicia et al [49], Demers et al [50], Freund et al [51], Johnson et al [52], Kerr et al [53], Krishnan et al [54], Lobo et al [55], Luo et al [56], Peters et al [57], Sarfo et al [58], Saywell et al [59], Silvera-Tawil et al [60], Sun et al [61], Xu et al [62], Lee et al [63], Mainali et al [64], Reeves et al [65], Requena et al [66], Cooray et al [67], Blanton et al [68], Caunca et al [69], Sureshkumar et al [70], Zhou et al [71], Vloothuis et al [72], English et al [73], Givon et al [74], Giachero et al [75], Lo et al [76], Andrades-González et al [77]; Demir and Gozum [78], De Simoni et al [79], Eriksson et al [80], Favilla et al [81], Firdaus et al [82], Firmawati et al [83], Gong et al [84], Kamwesiga et al [85], Lobo et al [86], Kechik et al [87], Pereira et al [88], Newland et al [89], Nichols et al [90], Siegel et al [91], Tsang et al [92], Lee et al [93], Olafsdottir et al [94], Olafsdottir et al [95], Juengst et al [96], ‌Lam et al [97], Lo et al [98]	52 (67)
Community functions and roles	Leonardi and Fheodoroff [3], Lobo et al [7], Gooch et al [9], Smythe et al [10], Sauvé-Schenk et al [12], Sauerzopf et al [15], Wright et al [23], Magwood et al [39], Aravind et al [99], Cameron and Gignac [100], Fu et al [101], Pindus et al [102], White et al [103], Norlander et al [104], Deutschbein et al [105], Millar et al [106], Berkeley et al [107], Murakami et al [108]	18 (23)
Online stroke community system	Smith et al [8], Smith et al [109], Reszel et al [110], Cruickshank et al [111], Thomas et al [112], Paul et al [113]	6 (8)
Stroke ecosystem	Feigin et al [14]	1(1)

Studies were conducted in a range of settings, as shown in Table 2. Eight articles, mostly review articles, did not focus on a specific country; of the other articles, most drew on stroke research conducted in high-income (developed) countries.

**Table 2.** Location of studies reported in the reviewed articles.

Study location	Reference list number	Number of papers (%)
Australia / Oceania	Lobo et al [7]^a^, Scrivener et al [48], Johnson et al [52], Saywell et al [59], Silvera-Tawil et al [60], English et al [73], White et al [103]	7 (9)
North America	Reszel et al [110], Demers et al [50], Krishnan et al [54], Mainali et al [64], Reeves et al [65], Blanton et al [68], Caunca et al [69], Favilla et al [81], Siegel et al [91], Aravind et al [99], Cameron and Gignac [100], Berkeley et al [107], Lee et al [93]	13 (17)
East Asia	Lee et al [63], Zhou et al [71], Lo et al [76], Gong et al [84], Tsang et al [92], Fu et al [101], Murakami et al [108], Lam et al [97], Lo et al [98]	9 (12)
South-East Asia	Luo et al [56], Xu et al [62], Firdaus et al [82], Kechik et al [87]	4 (5)
West Asia	Givon et al [74], Demir and Gozum [78]	2 (3)
South Asia	Sureshkumar et al [70]	1 (1)
Northern Europe	Lobo et al [7]^a^, Cooray et al [67], Eriksson et al [80], Norlander et al [104], Olafsdottir et al [94], Olafsdottir et al [95]	6 (8)
Western Europe	Sauerzopf et al [15], Wright et al [23], Smith et al [109], Kerr et al [53], Vloothuis et al [72], De Simoni et al [79], Deutschbein et al [105], Millar et al [106], Thomas et al [112], Paul et al [113]	10 (13)
Southern Europe	Requena et al [66], Giachero et al [75], Andrades-González et al [77], Pereira et al [88]	4 (5)
East Africa	Kamwesiga et al [85]	1 (1)
West Africa	Sarfo et al [58], Nichols et al [90]	2 (3)
South Africa	Smythe et al [10]	1 (1)
Worldwide/ not specified	Leonardi and Fheodoroff [3], Thompson et al [4], Smith et al [8], Gooch et al [9], Sauvé-Schenk et al [12], Feigin et al [14], Magwood et al [39], Camicia et al [49], Freund et al [51], Lobo et al [55], Peters et al [57], Sun et al [61], Firmawati et al [83], Lobo et al [86], Newland et al [89], Pindus et al [102], Cruickshank et al [111], Juengst et al [96]	18 (23)

a This study was conducted in more than one country.

Table 3 shows the methodological approaches reported in each article. The 19 review articles aside, empirical articles varied in their approaches: descriptive qualitative methods (including a range of approaches such as content analysis, grounded theory, and thematic analysis); randomized control trials; interventions; feasibility/usability (quantitative) studies; one prospective cohort quantitative study; and mixed methods studies. We also found that 3 articles reported co-design or consensus studies.

**Table 3.** Methodological approaches.

Study methodology used	References	Number of papers (%)
Review article	Leonardi and Fheodoroff [3], Thompson et al [4], Lobo et al [7], Gooch et al [9], Sauvé-Schenk et al [12], Feigin et al [14], Magwood et al [39], Camicia et al [49], Freund et al [51], Lobo et al [55], Peters et al [57], Sun et al [61], Firmawati et al [83], Lobo et al [86], Kechik et al [87], Newland et al [89], Cameron and Gignac [100], Pindus et al [102], Juengst et al [96]	19 (25)
Randomized control trial	Johnson et al [52], Saywell et al [59], Lee et al [63], Reeves et al [65], Zhou et al [71], English et al [73], Givon et al [74], Giachero et al [75], Lo et al [76], Favilla et al [81], Aravind et al [99], Fu et al [101], Lam et al [97]	13 (17)
Cluster Randomized Trial	Berkeley et al [107]^a^	1 (1)
Clinical trial	Scrivener et al [48]	1 (1)
Feasibility/usability (quantitative) study	Mainali et al [64], Caunca et al [69], Demir and Gozum [78]	3 (4)
Intervention study	Luo et al [56], Requena et al [66], Cooray et al [67], Vloothuis et al [72], Deutschbein et al [105], Olafsdottir et al [95], Paul et al [113]	7 (9)
Observational study (quantitative)	Demers et al [50], Sarfo et al [58], Firdaus et al [82], Siegel et al [91], Berkeley et al [107]^a^, Murakami et al [108]	6 (8)
Qualitative study	Smythe et al [10], Sauerzopf et al [15], Wright et al [23], Smith et al [109], Reszel et al [110], Krishnan et al [54], Xu et al [62], Andrades-González et al [77], De Simoni et al [79], Eriksson et al [80], Kamwesiga et al [85], Tsang et al [92], Norlander et al [104], Cruickshank et al [111], Thomas et al [112], Lo et al [98]	16 (20)
Mixed methods	Smith et al [8], Blanton et al [68], Sureshkumar et al [70], Gong et al [84], Nichols et al [90], Millar et al [106], Lee et al [93], Olafsdottir et al [94]	8 (10)
Co-design	Silvera-Tawil et al [60], Pereira et al [88]	2 (3)
Consensus method	Kerr et al [53]	1 (1)
Prospective Cohort (quantitative)	White et al [103]	1 (1)

a Article involves a secondary observational analysis of a cluster-randomized trial, so is listed twice.

### Data Synthesis

The reviewed articles were concerned with a range of different study respondents (Table 4a). Almost all articles focused on stroke survivors. Most were concerned with informal caregivers, often family members or friends; a similar but slightly smaller number of articles were concerned with community or social support. Health workers were the focus of approximately one-third of the reviewed articles, while a much smaller number of articles concerned policymakers and funding bodies. Some articles addressed multiple study populations.

**Table 4.** Summary of study respondents in reviewed articles.

Respondents	References	Number of papers (%)
Stroke caregivers (ie, family and friends)	Thompson et al [4], Lobo et al [7]^a^, Smith et al [8], Gooch et al [9]^a^, Smythe et al [10]^a^, Sauvé-Schenk et al [12]^a^, Sauerzopf et al [15]^a^, Wright et al [23]^a^, Magwood et al [39], Smith et al [109]^a^, Camicia et al [49]^a^, Freund et al [51], Kerr et al [53]^a^, Lobo et al [55], Sun et al [61]^a^, Xu et al [62], Cooray et al [67], Blanton et al [68]^a^, Caunca et al [69], Sureshkumar et al [70], Zhou et al [71]^a^, Vloothuis et al [72]^a^, English et al [73], Andrades-González et al [77], Demir and Gozum [78], De Simoni et al [79], Eriksson et al [80], Favilla et al [81], Firdaus et al [82], Firmawati et al [83], Gong et al [84], Kamwesiga et al [85], Lobo et al [86], Kechik et al [87], Pereira et al [88]^a^, Nichols et al [90]^a^, Tsang et al [92], Aravind et al [99], Cameron and Gignac [100], Fu et al [101], Pindus et al [102], Norlander et al [104], Thomas et al [112], Olafsdottir et al [94], Olafsdottir et al [95]^a^, Lo et al [98]	46 (60)
Health workers (ie, therapist and nurse)	Leonardi and Fheodoroff [3], Thompson et al [4], Lobo et al [7]^a^, Gooch et al [9]^a^, Smythe et al [10]^a^, Feigin et al [14]^a^, Sauerzopf et al [15]^a^, Magwood et al [39], Reszel et al [110], Camicia et al [49]^a^, Kerr et al [53]^a^, Silvera-Tawil et al [60], Sun et al [61]^a^, Mainali et al [64], Requena et al [66], Blanton et al [68]^a^, Zhou et al [71]^a^, Vloothuis et al [72]^a^, Pereira et al [88]^a^, Newland et al [89], Nichols et al [90], Millar et al [106], Lee et al [93], Olafsdottir et al [95]^a^	24 (31)
Community/society	Gooch et al [9], Smythe et al [10]^a^, Sauvé-Schenk et al [12]^a^, Sauerzopf et al [15]^a^, Wright et al [23]^a^, Smith et al [109]^a^, Kerr et al [53]^a^, Luo et al [56], Sarfo et al [58], Sun et al [61]^a^, Nichols et al [90]^a^, Murakami et al [108], Lee et al [93], Paul et al [113]	14 (18)
Funders	Feigin et al [14]	1 (1)
Policymakers	Feigin et al [14], Kerr et al [53]^a^	2 (3)

a These studies involved more than one respondent categories.

**Figure 3.**
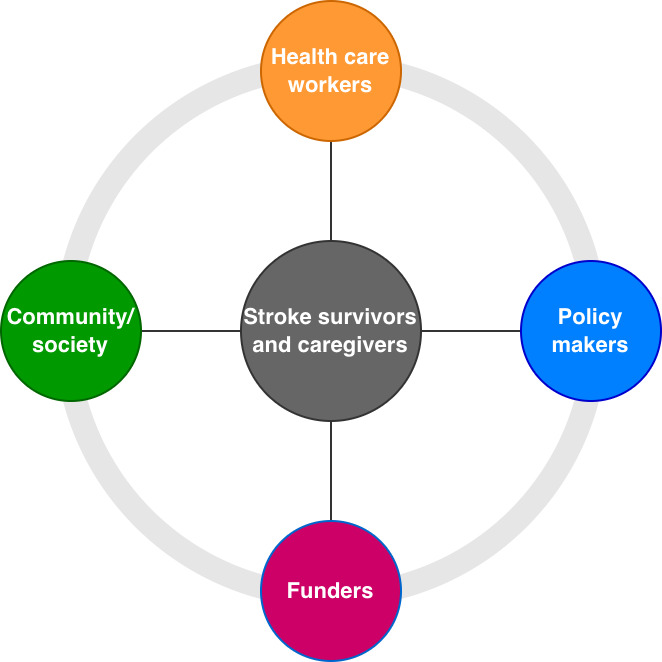
Actors who comprise the stroke health ecosystem.

The information presented in Table 4a highlights the different human actors involved in the provision of stroke-related health care and support. This stroke health ecosystem (Figure 3) consists of (informal) stroke caregivers, health workers, community members, or the broader society, funders, and policymakers.

Stroke caregivers are often a spouse or partner, family member, or friend who provides required support on a regular basis [7,8]. They are often untrained—although they may draw upon training they have received for other roles—and they are most often unpaid, although they may receive a small stipend for their care provision. Active engagement of stroke caregivers could reduce health care costs, reduce burnout, improve care processes, and improve stroke survivors’ outcomes [7]. Health workers offer separate and overlapping services designed to create a connection between stroke survivors and health care systems and facilitate the navigation of services to optimize stroke recovery, manage care transitions, limit social isolation, and help determine eligibility for services vital to stroke care [7]. They have received specific training relevant to their discipline (such as medicine, nursing, or allied health) or for the specific care tasks undertaken (such as completing a certificate or diploma in community care). They often receive a salary commensurate with their labor provisions. Community/society includes groups of individuals, organizations, or other types of networks and social circles [8,10]. Community/society may include neighbors or small business holders. Funders are agencies or philanthropists, including health insurance organizations, that provide funding for community-based stroke program design, development, and implementation [14]. Policymakers are health regulators who seek to translate the insights from funders and grassroots care providers into formalized regional, national, and international regulations [14].

### Community Support

All but 2 articles discussed some aspects of community support. “Community support” is an umbrella term that captures a range of support activities and processes (Table 5).

**Table 5.** Types of community support included in the reviewed articles.

Types of community support	References	Number of papers (%)
Informational support	Smith et al [8], Feigin et al [14]^a^, Reszel et al [110]^a^, Camicia et al [49]^a^, Johnson et al [52]^a^, Lobo et al [55]^a^, Reeves et al [65]^a^, Cooray et al [67], Blanton et al [68]^a^, Caunca et al [69], Sureshkumar et al [70], Lo et al [76]^a^, Andrades-González et al [77]^a^, Demir and Gozum [78], De Simoni et al [79]^a^, Eriksson et al [80]^a^, Favilla et al [81], Firdaus et al [82], Firmawati et al [83]^a^, Lobo et al [86], Kechik et al [87], Nichols et al [90], Siegel et al [91], Tsang et al [92], Cruickshank et al [111]^a^, Thomas et al [112]^a^, Olafsdottir et al [94]^a^, Lo et al [98]^a^	28 (36)
Emotional support	Smith et al [8], Smith et al [109], Blanton et al [68]^a^, Firmawati et al [83]^a^, Thomas et al [112]^a^, Olafsdottir et al [94]^a^, Olafsdottir et al [95]^a^, Juengst et al [96]^a^	8 (10)
Social support	Leonardi and Fheodoroff [3], Smith et al [8], Feigin et al [14]^a^, Reszel et al [110]^a^, Camicia et al [49]^a^, Freund et al [51]^a^, Johnson et al [52]^a^, Peters et al [57]^a^, Sarfo et al [58]^a^, Saywell et al [59], Silvera-Tawil et al [60]^a^, Sun et al [61]^a^, Xu et al [62], Lee et al [63]^a^, Lo et al [76]^a^, Andrades-González et al [77]^a^, De Simoni et al [79]^a^, Eriksson et al [80]^a^, Kamwesiga et al [85], Pereira et al [88], Cruickshank et al [111]^a^, Thomas et al [112]^a^, Olafsdottir et al [94]^a^, Olafsdottir et al [95]^a^, Lo et al [98]^a^, Paul et al [113]	26 (33)
Esteem support	Scrivener et al [48], Olafsdottir et al [94]^a^	2 (3)
Spiritual support	Smith et al [8]	1 (1)
Advice or knowledge	Smith et al [8], Feigin et al [14]^a^, Reszel et al [110]^a^, Camicia et al [49]^a^, Freund et al [51]^a^, Johnson et al [52]^a^, Lobo et al [55]^a^, Lee et al [63]^a^, Lo et al [76]^a^, Gong et al [84], Newland et al [89], Cruickshank et al [111]^a^, Olafsdottir et al [94]^a^, Olafsdottir et al [95]^a^	14 (18)
Tangible aid	Smith et al [8], Feigin et al [14]^a^, Scrivener et al [48]^a^, Camicia et al [49]^a^, Johnson et al [52]^a^, Kerr et al [53], Krishnan et al [54], Lobo et al [55]^a^, Luo et al [56], Peters et al [57]^a^, Sarfo et al [58]^a^, Silvera-Tawil et al [60]^a^, Sun et al [61]^a^, Lee et al [63]^a^, Mainali et al [64], Reeves et al [65]^a^, Requena et al [66], Zhou et al [71], Vloothuis et al [72], English et al [73], Givon et al [74], Giachero et al [75], Lo et al [76]^a^, Deutschbein et al [105], Lee et al [93], Olafsdottir et al [94]^a^, Olafsdottir et al [95]^a^, Juengst et al [96]^a^, Lam et al [97]	29 (38)
Involvement in decision-making	Camicia et al [49]^a^, Blanton et al [68]^a^	2 (3)
Not mention the type of community support	Thompson et al [4], Demers et al [50]	2 (3)

a These studies mentioned more than one type of community support.

Our review highlights that there are sometimes different meanings given to each type of support; thus, we defined each type of support by searching for similar meanings across studies. The reviewed articles indicate that the supports most needed by stroke survivors and caregivers are informational support, network/social support, and tangible aid (Multimedia Appendix 4 [3,4,8,14,48-61,63-98,105,109-113]).

Informational support has an educative element, consisting of ideas or suggestions for action, or providing sources of information or help. This information can help stroke survivors and their caregivers to reassess or redefine their situation, while also offering detailed information, facts, or news [23]. Emotional support is a nurturing support that helps individuals cope with the emotional consequences of a stressor [23,109]. Esteem support consists of compliments, provides agreement with the views of the recipient, and alleviates any feelings of guilt the recipient has about the situation [23,109]. Network or social support provides the stroke survivors or caregivers with access to people outside of their “inner circle” and reminds them that there are others who share similar experiences and are available for support [23,109]. Spiritual support is concerned with how people’s religious or spiritual beliefs can create a sense of connection to cope with their diseases [8,34]. In contrast, tangible aid involves directly observable and agentic support and can include offers to do a direct task or to take over a task from the stroke survivors while they are stressed, to join the stroke survivors in an activity, and offers or expressions of willingness to help; it may also include lending money to the stroke survivors and caregivers [23]. Tangible aid can also involve the provision of meals, undertaking cleaning, or completing other tasks [109]. Stroke survivors and caregivers also need involvement in decision-making on the model of care, such as treatment that should be done to ensure optimal recovery for the stroke survivors [7,102].

### Technological Interventions

A total of 60 of the 77 articles included in this review were concerned with a diversity of technological interventions aimed at supporting people after stroke. These included: (1) communication technologies (including emails or online videoconferencing); (2) community building, including the use of online forums; (3) well-being-related; for the purposes of health monitoring and management (such as sensors or wearable technology); or for the purposes of engaging with the world (such as via VR). Table 6 outlines these results.

**Table 6.** Technological interventions explored in the reviewed articles.

Technological intervention	References	Number of papers (%)
Mobile health (mHealth)	Thompson et al [4], Feigin et al [14]^a^, Camicia et al [49]^a^, Kerr et al [53], Lobo et al [55]^a^, Sarfo et al [58], Silvera-Tawil et al [60], Requena et al [66], Cooray et al [67], Zhou et al [71], Vloothuis et al [72], Andrades-González et al [77], Eriksson et al [80], Firdaus et al [82], Firmawati et al [83], Gong et al [84], Lobo et al [86], Kechik et al [87], Pereira et al [88], Nichols et al [90], Siegel et al [91], Olafsdottir et al [94]^a^, Olafsdottir et al [95]^a^, Juengst et al [96], Paul et al [113]	25 (32)
Electronic health record (EHR) or electronic medical record (EMR)	Deutschbein et al [105]	1 (1)
Electronic mail (email)	Cruickshank et al [111]^a^	1 (1)
Web-based system	Leonardi and Fheodoroff [3], Feigin et al [14]^a^, Reszel et al [110], Camicia et al [49]^a^, Freund et al [51], Lobo et al [55]^a^, Reeves et al [65], Blanton et al [68], Caunca et al [69], Sureshkumar et al [70], Demir and Gozum [78], Favilla et al [81]	12 (16)
Online forum	Smith et al [8], Smith et al [109], De Simoni et al [79], Thomas et al [112]	4 (5)
Telehealth or telerehabilitation or teleconsultation or telestroke	Saywell et al [59], Mainali et al [64], Lo et al [76], Lam et al [97]	4 (5)
Video-guided exercise app	Scrivener et al [48], Camicia et al [49]^a^, Givon et al [74]^a^, Newland et al [89]	4 (5)
Communication technology such as video conferencing (ie, Zoom) and instant messaging (IM) app	Tsang et al [92], Cruickshank et al [111]^a^	2 (2)
Virtual reality (VR) or augmented reality (AR)	Johnson et al [52], Krishnan et al [54], Luo et al [56], Sun et al [61], Lee et al [63], Givon et al [74]^a^, Giachero et al [75], Lo et al [98]	8 (10)
Sensors or wearable technology	Demers et al [50], Peters et al [57], Sun et al [61]^a^, English et al [73], Kamwesiga et al [85], Lee et al [93], Olafsdottir et al [94]^a^, Olafsdottir et al [95]^a^	8 (10)
General platform use	Lobo et al [7], Gooch et al [9], Smythe et al [10], Sauvé-Schenk et al [12], Sauerzopf et al [15], Wright et al [23], Magwood et al [39], Xu et al [62], Aravind et al [99], Cameron and Gignac [100], Fu et al [101], Pindus et al [102], White et al [103], Norlander et al [104], Millar et al [106], Berkeley et al [107], Murakami et al [108]	17 (22)

a These studies explored more than one technological intervention.

### Key Actors in Providing Technology-Mediated Support

Findings on community support and technological interventions were then compared across the different population and cohort groups. This often involved actors—individuals, groups/communities, and agencies who played an important role—beyond those who were study respondents, as can be seen in Table 5. Rather than summarizing Tables 4-6, Table 7 illustrates that different cohorts held different priorities in terms of support needs and the digital interventions used. Importantly, stroke caregivers were both providers and recipients, as is reflected in Table 5 and discussed below. Most technologies are developed to provide support from stroke caregivers, online communities/society, and health workers. A matrix approach provides more granular insights into the elements and interrelationship between elements of a stroke community ecosystem.

**Table 7.** Mapping actors, type of community support and supporting technology.

	Informational support	Emotional support	Social support	Esteem support	Spiritual support	Advice or knowledge	Tangible aid	Involvement in decision-making	Does not mention the type of online community support
Stroke caregivers (ie, family, friends)	Online forum [8,79,112], mHealth^a^ [14,49,55,67,77,80,82,83,86,87,90,91,94], web-based system [14,68-70,81,110], VR/AR^c^^d^ gaming [98], IM^e^ app [92]	Online forum [8,109,112], web-based system [68], mHealth [83,94-96]	Online forum [8,79,112], VR/AR [63,98], mHealth [14,49,77,80,88], mobile phone [85], web-based system [3,51,110]	Video-guided exercise app [48], mHealth [94]	Online forum [8]	Online forum [8]	VR/AR [54,61,74,75], mHealth [53,55,71,72,94-96], sensors or wearable technology [73,94,95], telehealth [76,97], video-guided exercise app [74], online forum [8], general platform use [23,107]	Web-based system [49,68]	mHealth [4], sensors or wearable technology [50]
Health workers (ie, therapist, nurse)	Online forum [112], VR/AR [52], telehealth [76], web-based system [65,68,78], mHealth [90,91], general platform use [10,12,39,101,102,105,106]	General platform use [101,106]	Online forum [112], wearable technology [85], mHealth [88], general platform use [7,10,15,99,106]	—^f^	—	VR/AR [52,63], mHealth [14,49,55,84,94,95], sensors or wearable technology [94,95], telehealth [76], video-guided app [49,89], web-based system [51,110], general platform use [101]	VR/AR [52,63,74,75], mHealth [49,53,58,66,71,72,94,95], video-guided exercise app [48,74], sensors or wearable technology [57,93-95], web-based system [65], telehealth [64,76,97], general platform use [107]	General platform use [7,102,106]	—
Community/society	Email [111], video conferencing [111], online forum [8,112], web-based system [65,68,69,110], mHealth [90], general platform use [7,9,10,23]	Online forum [8,109,112], general platform use [23,102]	Video conferencing [111], online forum [8,112], VR/AR [52], mHealth [58,60,94,95,113], sensors or wearable technology [57,61,85,94,95], telehealth [59,76], general platform use [9,10,12,15,23,102-104,106,108]	General platform use [12,23]	Online forum [8]	Email [111], video conferencing [111], online forum [8]	mHealth [53,58,60], telehealth [97], VR/AR [56,74,75], web-based system [55,65], video-guided exercise app [74], sensors or wearable technology [61], general platform use [23,107]	General platform use [102,106]	Sensors or wearable technology [50]
Funders	—	—	—	—	—	—	EHR/EMR^g^^h^ [105] mHealth [14]	—	—
Policymakers	—	—	—	—	—	—	mHealth [14,53], EHR/EMR [105]	—	—

b mHealth: mobile health.

c VR: virtual reality.

d AR: augmented reality.

e IM: instant messaging.

f Not available.

g EMR: electronic medical record.

h EHR: electronic health record.

### Stroke Survivors’ and Caregivers’ Support Requirements in the Online Community

#### Informational Support

Thomas et al [112] found that there is still a knowledge gap between health workers and stroke survivors and caregivers, which is a major contributor to burden for stroke survivors and caregivers [69]. As a result, they search for information from online communities to provide informal explanations and reassurance beyond that provided by the health workers [112].

The information sought is wide-ranging. Stroke survivors and caregivers require information on life after stroke, including about the underlying diseases, activities of daily living, healthy behaviors, medical information, treatment options, home-based exercises, functional skills training, and symptoms, as well as risk factors for stroke, such as ambulation and fall risk [14,23,69,70,78,79,82,83,86,87,90]. Blanton et al [68] found that providing information via short videos that engaged stroke survivors and caregivers in practicing physical tasks in the home environment would be welcomed.

The informational needs of stroke survivors and caregivers extended to mental health and well-being issues, including stress management, self-care, and practical fatigue management strategies [69,112]. These findings echo those of Lo et al [76] and Firmawati et al [83], who found that survivors and caregivers need information related to stroke care: nutrition, exercise, medications, community services, peer-sharing, and expert advice. They saw videos as appropriate for enhancing caring skills and confidence, and offering support to caregivers [81,83]. Narrating the story of stroke was seen as significant in prompting different forms of information sharing. Wright et al [23] and Smith et al [109] argued that offering stroke survivors an opportunity to share their rehabilitation journey story acted as a way to request (and offer) support from online communities.

Informational needs extend beyond the stroke survivor and their caregiver to other actors in their community. Clinical information is required by health workers for monitoring the health of stroke survivors. While this can occur through clinical and follow-up assessments or surveillance data, these can be complicated to achieve in some settings [14,67]. The source of required information should not only come from related actors such as health workers or health facilities but should also be integrated with medical record data [67]. Information principles should be well-organized, complete, and necessary, with helpful information provided in an accessible format, and including adequate resources [69].

#### Emotional Support

The reviewed research indicated that while stroke was seen as a physical illness, stroke survivors’ emotions were affected alongside their physical function [106], thus emphasizing the need for emotional support for stroke survivors and caregivers. However, as found by Pindus et al [102], emotional support was lacking for stroke survivors and caregivers. For stroke caregivers, the required emotional support included acceptance of caregiving situations, depressive symptoms, and stress management [69,83,109]. Behavioral and cognitive issues—especially memory problems and resistance to accept help—experienced by the stroke survivors were of concern to the stroke caregivers as well as survivors themselves [69].

Through their analysis of an online community for pediatric stroke survivors, Wright et al [23] defined emotional support as including relationships, physical affection, confidentiality, sympathy, understanding or empathy, and encouragement. The importance of closeness was emphasized [23], including through offering physical affection (including via hugs and kisses) that leads to positive emotions [23,101,112]. Emotional support that facilitates individual positive emotions was noted to elevate one’s overall health level and reduce negative emotions such as anxiety, fatigue, and depression [101,112]. Also important was the need for all support actors to keep stroke survivors’ problems or difficulties confidential [23] and was especially important for online support communities. Gender may moderate these priorities as reported by Smith et al [8], who noted that women are likely to seek both emotional and informational support compared with men.

Online communities could also provide a platform to express sorrow or regret for the situation, and to seek reassurance or strategies for dealing with ongoing symptoms. They also allowed stroke survivors and caregivers to express their struggles in attempting to find a cause or express their difficulties in understanding the situation [23]. At the same time, by providing a platform to disclose and acknowledge similar experiences in a way that conveys understanding, online communities provided stroke survivors and caregivers with hope and confidence [23]. Empathy for stroke survivors was often given by online communities, with emphasis placed on loneliness after stroke, strategies to fight the urge to give up on recovery, recognition of the symptoms experienced, and articulation of practical difficulties [23].

#### Esteem Support

Esteem support was defined as distinct from emotional support by Wright et al [23] as a form of recognition (for example, of the stroke survivor’s contribution to a community) as well as to validate people’s recovery processes. Sauvé-Schenk et al [12] found that identifying social service and community resource needs was important for tailoring stroke recovery interventions to stroke survivors’ and caregivers’ individual situations.

Esteem support also includes having the support to build esteem in others. While home-based exercise can lead to good functional results, esteem support can enhance these further. Olafsdottir et al [94,95] found that providing caregivers with information and knowledge on how to motivate the stroke survivor can empower them to become more willingly involved in the rehabilitation process at home and thus to support and motivate their stroke survivor to exercise and be more physically active. As reported by Olafsdottir et al [95] and Scrivener et al [48], community-dwelling stroke survivors found home-based exercise and physical activities more fun and less tedious with more variety in exercise and training options.

The categories of esteem support include compliments, validation, and relief of blame. Relief of blame functioned as support by reassuring a stroke survivor that they were not a burden and everyone needs support at some point [23]. Wright et al [23] also found that compliments were given to motivate stroke caregivers, and to congratulate people for joining the community. Validation was given when stroke survivors had been through bad experiences such as missed or delayed diagnoses, poor awareness of stroke, isolation, feeling tired, and wanting live chat rooms [23].

#### Social Support

The reviewed articles emphasized that it was not only stroke survivors who faced challenges or burdens after stroke, with many caregivers describing similar conditions. Most caregivers were unprepared for their caregiving role, particularly given the immediate need to provide care following their family member’s stroke [7]. Norlander et al [104] found that people’s abilities to overcome challenges and to adapt their behavior and attitude played a critical role in social participation and integration after stroke. Social participation refers to a person’s engagement in activities with family, friends, peers, or community members [88]. Social integration, in contrast, is specifically concerned with the linkages between stroke survivors and caregivers with other members of their community [88]. Providing network or social support is a long-term process that constantly needs to be balanced against other priorities in life. Thus, Norlander et al [104] urged that network involvement need not only emphasize supporting each other, but also should emphasize people’s motivations to be engaged in the network.

Social support was generally present in forums where messages expressed a sense of camaraderie, reiterating that members of the forum were there to support each other [23]. Social support activities could also be engaged in for pleasure, relaxation, or other emotional satisfaction, thus increasing stroke survivors’ and caregivers’ well-being [104]. At the same time, these social support activities were found to reduce depressive symptoms and improve functional recovery and survival after stroke [104]. By using social support in the community, stroke survivors and caregivers could also get information, knowledge, and resources related to stroke [88,102,111].

Social support often operated alongside esteem support, as could be seen where peer connection provided motivation to seek out and engage with different health workers [111]. Social support could also assist stroke survivors to use tools for physical activities or exercise in order to become familiar, comfortable, safe, and encouraged to finish their activities or exercises [94]. Olafsdottir et al [94] described examples of using tools on the proper use of a wheelchair in various scenarios—such as boarding a bus, parking a wheelchair, exiting a bus, using an elevator, and general maneuvering—as especially useful for stroke survivors and caregivers, thus linking with tangible aid (discussed below).

#### Spiritual Support

Our search identified limited studies [8,62] regarding prayer support, which we then combined with spiritual support to create a single category. Smith et al [8] define prayer support as a subcategory of spiritual support to improve the well-being, spiritual, or energetic state of the stroke survivor; however, prayer support does not have a material outcome (thus contrasts with action-facilitating forms of support, as in tangible aid). Stroke survivors wanted to be close with their religious community, such as the prayer group to which they belonged. Prayer or spiritual support includes providing and seeking prayers, spiritual blessings, positive karma, and warm thoughts in the community [8], and was given by writing comments for the online community [8]. Xu et al [62] described spiritual or prayer support as a form of healing, and others felt the need to retain a positive approach and not become a burden.

#### Advice or Knowledge

Stroke survivors and caregivers required advice or knowledge about stroke rehabilitation, specifically that provided by health care workers in the community on physical activity or exercise [14,94]. They also appreciated advice based on the stroke experiences of peers in the community [76]. Stroke caregivers expressed their need for education on how they could provide support with exercise and physical activity for the stroke survivors [94]. Stroke survivors and caregivers also needed advice on knowledge repositories or management tools, such as internet search engines, to be able to interact in the online community [55].

Advice and knowledge needed to be localized. Aside from text messages, Gong et al [84] found that health workers’ voice messages using local dialects related to stroke advice or knowledge could prevent stroke among people with low education levels in resource-limited settings. Factors including voice message structure, language, complexity and relevance, and repetition all influenced stroke survivors’ and caregivers’ acceptance of using voice-based apps [84]. Short video apps could be used by nurses to provide stroke education [89]. Important factors in providing stroke education videos related to the videos’ frequencies, the ideal length of video delivery, and the need for repetitive opportunities to review educational stroke information [89].

#### Tangible Aid

Tangible aid for stroke survivors and caregivers was shown to consist of financial support (particularly through loans), and offering to take over tasks [23]. Multiple articles also identified that tangible aid could include the technologies and technical devices needed by stroke survivors and caregivers to improve their self-management [48,52,54,63,74,75,94]. Tangible aid was also facilitated via the provision of technology-based rehabilitation programs, which could provide a unique platform for stroke survivors who were unable to obtain rehabilitation services at professional institutions due to insufficient medical resources, economic disadvantage, and access issues (including inconvenient traffic) [75].

#### Involvement in Decision-Making

Five of the reviewed articles identified that the limited knowledge of stroke survivors and caregivers meant that community involvement in decision-making was sometimes necessary for certain actions such as taking certain medicine or treatment [7,49,68,102,106]. For example, Pindus et al [102] described that stroke survivors need help with their treatment and exercise planning, which enabled timely decisions for increasing their quality of life. Online communities facilitated the provision of informational support and advice, which could help stroke survivors and caregivers to improve their problem-solving and decision-making [102], such as about which health care disciplines to engage for particular issues.

Decision-making regarding treatments and exercises was identified by Millar et al [106] as needing to be shared equally by stroke survivors and their caregivers with health workers, who could advise on how to deal with issues or concerns as they arose. Nurses, for example, were not necessarily the most appropriate health workers to consult for medication management. In addition, the provision of prehospital screening and the development of stroke severity tools by online platforms could enhance stroke survivors’ decision-making to ensure they receive the most appropriate level of care [49].

#### Outlining an Online Community Ecosystem for Stroke Survivors and Caregivers

**Figure 4.**
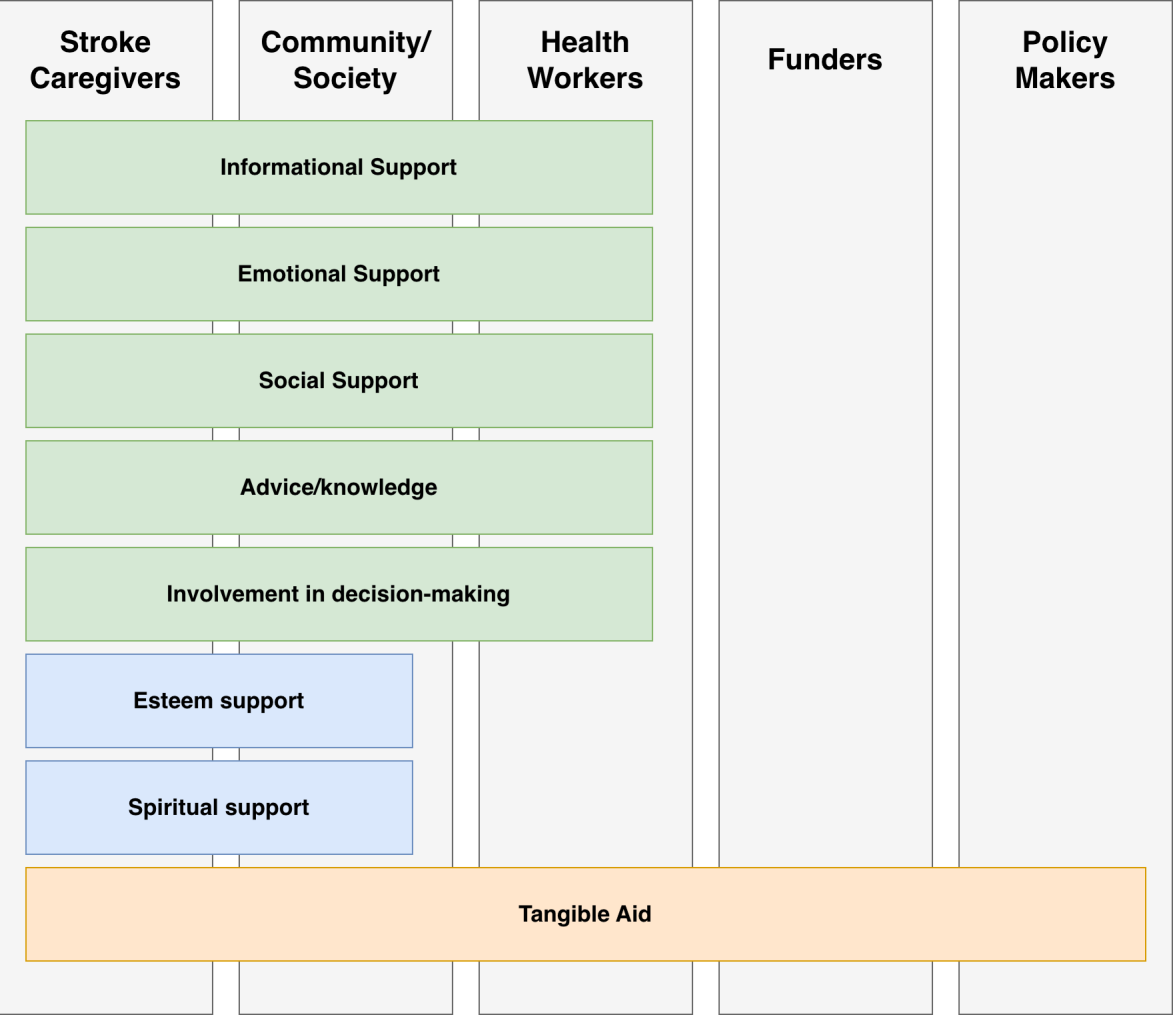
The online community ecosystem for stroke survivors.

Figure 4 describes a community ecosystem for stroke survivors that we have developed based on the reviewed literature. Of particular note, each layer of the ecosystem includes forms of support that are also provided through the lower layers.

Our review findings indicate that stroke caregivers and community provide all types of support, and thus comprise the core support team for most stroke survivors. Health workers are also important, providing informational support, emotional support, social support, advice/knowledge, tangible aid, and involvement in decision-making. Health workers—such as nurses, physical therapists, occupational therapists, and speech-language pathologists—were commonly involved in community-based interventions to facilitate the necessary support to optimize stroke recovery [7,107]. Among health workers, nurses often served as coordinators of care and offered direct delivery of a range of interventions in the home and community settings [7,49]. This, combined with their capacity for continuous care, positioned nurses as critical health worker actors who are uniquely positioned to improve stroke caregivers’ knowledge and assist stroke survivors [7,49].

Nurses play a key role both in acute care settings and postdischarge. Camicia et al [49], for example, emphasized the involvement of community-based health workers in providing longer-term support for stroke survivors following their transition home after a stroke. The community operates both as a destination of the recovery pathway and also as a partner that can help address the ongoing and often-unmet needs experienced postdischarge [49]. Partnerships between health services and the voluntary sector can offer innovative solutions to improve stroke survivors’ recovery care [49]. Partnerships are especially important in LMIC due to the lack of community rehabilitation services; in such settings, stroke prevention services can be provided by wider community networks [14].

Online health communities can contribute to supporting stroke survivors and caregivers by providing informational support, emotional support, and discussing their conditions in an online forum [23,112]. Based on Smith et al [8], stroke survivors who received prayer-related words in online health communities experienced lower negative emotions and better well-being, possibly because religious coping mechanisms allowed them to experience less fear of death, place their trust in God, and view their illness experiences with more positivity. However, our review highlights that there remain limited studies that analyze esteem support, spiritual support, and involvement in decision-making. Most studies focused on stroke survivors’ and caregivers’ provision and receipt of informational support, advice/knowledge, and tangible aid for stroke physical recovery.

#### Technologies Used to Support Stroke Survivors and Caregivers in the Online Community

Technology-based interventions in the online community have been recommended to better meet the needs of stroke survivors and caregivers [69]. Promising technologies to meet these needs and support stroke survivors and caregivers identified in this review (Table 4c) provide advantages such as increasing technological literacy, asynchronous communication, use of multimedia platforms, and the potential to reduce disparities by increasing the reach of resources [69]. Most features used in technology-based interventions relate to rehabilitation and self-management for supporting changes in health behaviors, such as increasing physical activity [113]. Kerr et al [53] found the priorities for stroke rehabilitation systems are broad and include: access to technologies, ease of use, awareness of available technologies, technologies focused on function, supported self-management, user training, evidence of effectiveness, value for money, knowledgeable staff, and performance feedback.

Health workers were also shown to use similar technologies as stroke caregivers [61]. Our review showed that stroke caregivers used most technologies to provide support, except electronic health record/electronic medical record, email, digital health technology, and communication technologies such as video conferencing and IM apps. They also used technologies to search for information and tangible aid. mHealth, online forums, and web-based systems were the most used technologies by health workers, and were used by health workers to provide information, advice/knowledge, and tangible aid for stroke survivors’ rehabilitation processes [94,95,112]. Through those technologies, health workers could also easily monitor the progress status of stroke survivors’ health [48]. The community could provide all types of support to caregivers using similar technologies as health workers.

Since online forums could be used for 2-way communications, stroke caregivers also provided informational, emotional, and social support [79,109,112]. The online forum provided an open space to ask questions and share thoughts, as well as expressing comfort at reading the stories of other stroke survivors or caregivers, whilst at the same time stating their gratitude for receiving valuable assistance in a time of need [109].

mHealth and web-based systems were used to get and provide informational support, tangible aid, and involvement in decision-making for stroke caregivers. Camicia et al [49] argued that mHealth and telehealth or telerehabilitation could be used with other supporting tools to enhance the provision of care for stroke survivors. For example, clinicians could send digital education, community resources, tasks to complete (eg, home assessment), and surveys (eg, caregiver assessment) directly to the caregiver’s mHealth for timely evaluation and provision of treatment. Telerehabilitation refers to the use of any telecommunication modality (eg, telephone, videophone, audio-video conference) for the delivery of rehabilitation services at a distance [97].

Providing tangible aid to stroke caregivers and survivors offered a very clear purpose for technologies. Gooch et al [9] and Olafsdottir [95] described how digital health technologies—such as VR, robotics, sensors, messaging platforms, or audio-video platforms—could be used for physical stroke rehabilitation. Video-guided physical rehabilitation—largely via exercise programs with a self-management approach—was identified as a feasible and accepted set of interventions for people with stroke where health workers could provide advice, education about physical activity, and activities to enhance the skills required for self-management of physical activity [48]. Video-guided exercise could also provide esteem support to stroke caregivers and survivors by providing ongoing motivation and tips to keep their rehabilitation interesting [48]. VR/augmented reality and sensors could also be used for supporting the physical rehabilitation process for stroke survivors [52,63]. VR/augmented reality and sensors could be connected to an online platform for collecting stroke survivors’ information and providing timely feedback to stroke survivors’ and therapists [52].

Articles relating to social support from the community focused on potential improvements for existing and future technologies. Smith et al [8] argued that stroke technologies’ functionalities should always give stroke survivors and caregivers autonomy and agency, including the ability to approve or decline help offers from the community. Community-based health workers could provide training for stroke survivors and caregivers by using text messages, educational videos, and a workbook [14,49,68]. Criteria for health workers to use mHealth in assessing the appropriateness of mHealth apps were design, information/content, usability, functionality, ethics, security, privacy, and user-perceived value [96]. A key challenge for self-assessment and self-management using mHealth is keeping the stroke survivor and caregivers sufficiently engaged in the processes that are often repetitive, like answering questions about their status or completing home exercise programs; thus, reminders, notifications, and gamification features are deemed important to be provided in the mHealth [96].

Leonardi and Fheodoroff [3] found that internet-based or web-based systems could allow better exchange of information or stroke surveillance by involving communities, thus allowing data in real time and improving data quality [14]. Reszel et al [110] found that an internet-based system could be used to plan community-based exercise programs for people with stroke, such as conducting a thorough community assessment and developing referral pathways from the community. However, Sureshkumar et al [70] found that a major concern of internet-based systems was internet connectivity, especially in countries that do not have a stable internet connection.

Most online health communities provide support toward healing rather than health promotion [8]. These online communities help stroke survivors and caregivers through the rehabilitation process with the goal of improving functioning and prolonging the lives of stroke survivors [14].

Participatory design or co-design is often used when interactive technologies are being developed [95]. Smith et al [8] and Kerr [53] advocated for the use of participatory design that could engage all related actors, including technology developers and users, to design compassionate technology for facilitating social support for patients with life-threatening illnesses. This participatory approach also includes consideration of trust as a factor that affects sensitive disclosures and use of technology during health crises; Smith et al [8] found that stroke survivors and caregivers already trust app-based transportation and financial services, which could be leveraged to generate better health outcomes.

Communication technologies such as telephone calls, short message services, or WhatsApp messages could also be integrated into the online community for easier exchange of information and notification of new information/discussion in the community [78,80,85,92]. Cruickshank et al [111] explained that video conferencing and emails could be used in virtual community-based stroke programs to optimize participant experiences and outcomes. A reliable sender, an accepted form of the message dispatch, an optimal timing based on stroke survivors’ daily routine, and simple but relevant key messages were essential for the acceptance of message-based interventions [84]. Specifically, for voice messages, the most important factors that should be considered are the speed of audio playback, the pattern of repetition, and the use of dialect that may also improve audiences’ understanding of the message contents [84]. Based on Kamwesiga et al [85], follow-up calls, text messages, and reminders can be used to support the rehabilitation process after stroke.

Innovative and emerging technologies, such as generative artificial intelligence (AI), could also be explored to help online communities get more information and support. While Jiang et al [114] found that AI is mostly still under exploration in research on stroke early detection and diagnosis, treatment and outcome prediction, and prognosis evaluation, an AI-driven chatbot is available and can be embedded into virtual community-based stroke programs.

Funders (ie, health regulators, nongovernment organizations, and private organizations) and policymakers (ie, health regulators) provide tangible aid to stroke survivors in the community [14]. Policymakers used mHealth to support stroke survivors’ rehabilitation processes [53] by promoting the use of technologies in stroke rehabilitation. Policymakers are responsible for defining policies regarding the use of technology that can support online communities in supporting stroke survivors and caregivers [14,53]. They should engage with online communities, especially in providing health information and education related to stroke [14]. Policymakers also need to create a health ecosystem for managing stroke in a comprehensive and sustainable manner, where the online community is one of the actors involved in this ecosystem [14,53].

## Discussion

### Overview

Stroke recovery is a process that should be monitored and maintained for survivors and caregivers. This scoping review found that the key actors supporting stroke survivors are caregivers, the local community/society, and health workers. However, as stroke survivors face many difficulties during the rehabilitation process, it is important to identify innovative and scalable support solutions. Online platforms offer promising ways forward in the development of such solutions, and thus, the reviewed research emphasized the importance of online communities for stroke survivors and caregivers. The functions of online communities are to provide informational support, emotional support, esteem support, social support, spiritual support, advice/knowledge, tangible aid, and involvement in decision-making.

Even though technology has been shown to effectively connect stroke survivors and caregivers to online communities, there is a lack of integrated, available technology. As this review illustrates, stroke research related to the community and technology is underdeveloped. Therefore, this study provides insights for health regulators, health providers, health application developers, stroke survivors, and caregivers, as well as the community, to guide the provision of digital health technologies that could improve health outcomes. Our findings emphasize the importance of involving the online community in developing digital health apps for stroke survivors and caregivers to best support stroke recovery.

### Principal Findings

Our review identified only 3 studies [53,60,88] that actually engage with stroke survivors and their caregivers to develop the technologies used in the stroke community, yet one of these [53] included stroke survivors and caregivers as part of a broader “user community’s” priority setting activity. However, the importance of engagement is highlighted by a number of studies. From their user experience workshops with stroke survivors and health workers, Silvera-Tawil et al [60] found that there is a need for consumer-facing apps to integrate wearable activity trackers and a clinician web portal to support secondary prevention of stroke (ie, monitoring health and lifestyle measures). Pereira et al [88] conducted a workshop with stroke survivors, caregivers, and health workers to (1) explore the needs, concerns, motivations, and strategies for self-management support after stroke, (2) define the design principles based on social cognitive theory, and (3) develop and test a prototype using questionnaires to analyze usability. They found the need for personalized support for stroke management.

The design outcome from user-centered design should focus not only on the technology, but also on the health care outcomes. Lobo et al [55] and Lobo et al [86] suggested using user-centered design or participatory design to better understand the needs and respond to issues defined by users within the online community. In addition, Lobo et al [86], Kechik et al [87], Pereira et al [88], Siegel et al [91], and Juengst et al [96] suggested engaging stroke survivors and their caregivers in building apps to best meet their needs. In user-centered design, iterative processes—such as those generated by conducting workshops, focus group discussions, storyboards, games, etc—are required to better create technologies for intended users to support their daily activities [55,88]. Furthermore, these iterative processes may also help researchers or software developers to identify possible usability issues that may impact the interactions between the user and the technology [55,68,86].

Participatory design includes 2 principal values—participation and democracy—by involving several individuals with diverse knowledge and experiences [55]. Kerr et al [53] and Blanton et al [68] proposed the principles that should be implemented for stroke technologies, such as in the rehabilitation process, namely ease of use, evidence of effectiveness, access, and value for money to enhance the technology adoption by users (ie, therapists, stroke survivors, and caregivers). Stroke digital apps should be easy to use for end users, where they could use the apps with one hand and without too much trouble [53,55,68,82]. Any app can provide value for money if the end users feel the benefits of using the app [53].

Shaw [115] found that users’ technology confidence was positively associated with system usability. Shaw [115] described that digital inclusion, access to technology, and digital literacy were necessary to ensure universal access to and meaningful engagement with technology. Although technology infrastructure availability and accessibility are nationwide initiatives and policies, the online communities and health workers play a vital role in promoting digital equity, serving as educators, advocates, and digital navigators, such as guiding stroke survivors and caregivers through the complexities of the technology [115]. Multimedia Appendix 4 [3,4,8,14,48-61,63-98,105,109-113] summarizes technologies used to support stroke survivors in the community in the reviewed articles.

### Limitations

Several factors shaped this review, which only includes English-language journal articles. We focused specifically on stroke, and by excluding articles that included stroke as part of a broader focus on “disability or health,” we may have missed articles. Another limitation was related to terminology—an ever-expanding field. We focused on a subset of terminologies on the forms of technology; it is possible that some newer or less common technologies were not captured in our literature search. Similarly, online community support was also used as a broad umbrella term; some articles did not explicitly map the type of support for each actor, which meant that we inferred the types of support from descriptions given in the full-text article screening and extraction.

### Future Directions

Future research could analyze the required functionality of technologies that should be implemented in the health ecosystem. Advanced functionalities that could be integrated with emerging technologies, such as AI, could also be enhanced for stroke digital health technologies. Providing user-centered design and apps for stroke survivors and caregivers could increase the adoption of digital health technologies specifically for the recovery process of stroke survivors (ie, rehabilitation and self-management) [53,55]. Information through digital technologies should be personalized and delivered at appropriate times (such as medication delivery, self-monitoring, and so on) [55].

Cultural differences and socioeconomic factors remain underexamined in the reviewed articles. Future research should examine how these factors could influence the community and technology engagement, as well as how technologies supporting the online community are shaped. Smith et al’s [8] finding that gender may also influence health information-seeking behavior provides an important future direction by highlighting the importance of exploring stroke survivors’ and caregivers’ demographics to analyze in detail their information-seeking behaviors.

This review did not include social media as a specific source of support, although this remains a site for further exploring informal support, particularly in low-resource settings, where other technologies may be limited or unavailable. Future research should seek to understand how stroke survivors and caregivers access support in such settings, where a lack of health workers influences stroke recovery [14]. Finally, there is still limited research that explains in detail the role of funders and policymakers in the online stroke community; thus, future work could explore the strategic actors that could manage the sustainability of the stroke digital health apps in the online stroke community.

### Conclusions

**Figure 5.**
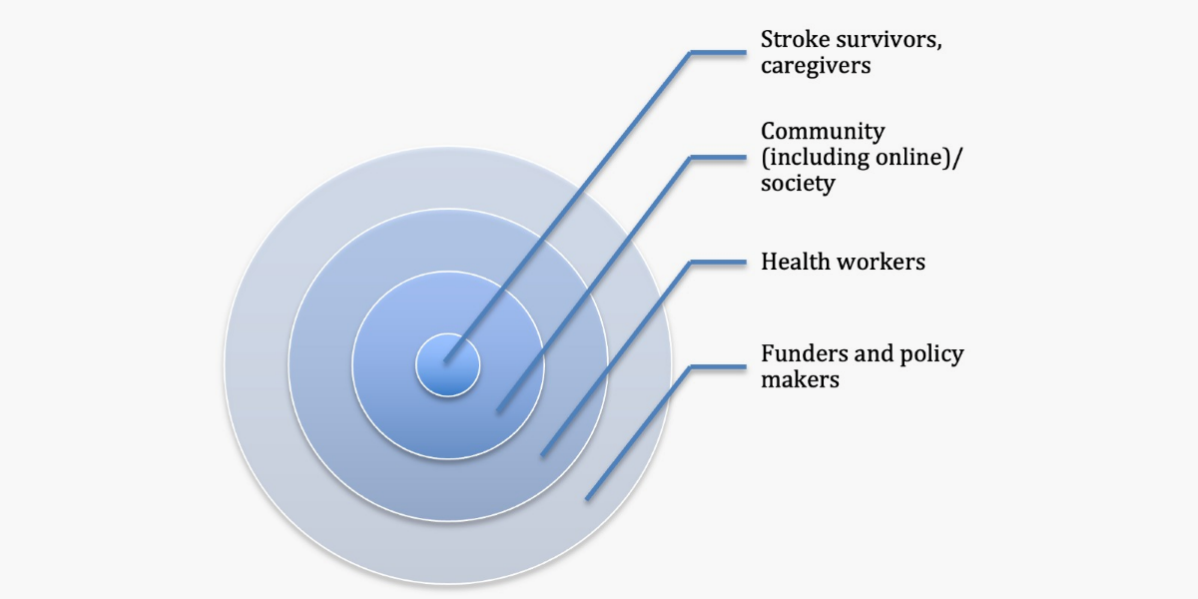
An expanded stroke ecosystem.

This review found that the online community plays an important role for stroke survivors and caregivers in their rehabilitation and recovery process and should be considered as part of a stroke ecosystem, in addition to stroke survivors, caregivers, health workers, community/society, funders, and policymakers (Figure 5).

Online communities provide diverse forms of support, including informational support, emotional support, esteem support, social support, spiritual support, advice or knowledge, tangible aid, and involvement in decision-making. They therefore play an integral role in this new, expanded stroke ecosystem and offer the potential to connect to and communicate with stroke survivors and caregivers. As technologies are constantly evolving, emerging technologies such as AI may extend this ecosystem in the future by addressing stroke early detection and diagnosis, treatment, and outcome prediction and prognosis evaluation.

## Supplementary material

10.2196/71190 Multimedia Appendix 1 Search strategy result.

10.2196/71190 Multimedia Appendix 2 Data chart item (rev 8).

10.2196/71190 Multimedia Appendix 3 Journals cited (rev 8).

10.2196/71190 Multimedia Appendix 4 Technologies used to support stroke survivors in the online community.

10.2196/71190 Checklist 1 PRISMA-ScR checklist.

## Abbreviations

AI: artificial intelligence
LMIC: low- and middle-income country
mHealth: mobile health
PRISMA: Preferred reporting items for systematic reviews and meta-analyses
PRISMA-ScR: Preferred reporting items for systematic reviews and meta-analyses extension for Scoping Reviews
VR: virtual reality
