# Supplementary material for: Online Community Support for Stroke Survivors and Caregivers: Scoping Review
Source: J Med Internet Res. 2026 Apr 29;28:e71190. doi: 10.2196/71190 (PMC13127857; doi:10.2196/71190)
Supplement: Multimedia Appendix 1 [file jmir-v28-e71190-s001.docx]

**Search Strategy Results**

Search date: October 7, 2025

| **Search term**   - Limits: Published since January 2015 to June 2025, Published in English | **PubMed** | **Proquest** | **Scopus** | **Web of Science** | **ScienceDirect** |
| --- | --- | --- | --- | --- | --- |
| **Concept 1: Stroke**  PubMed search terms:  stroke*[tiab] OR "cerebrovascular accident"[tiab] OR "post-stroke"[tiab] OR "stroke survivor*"[tiab] OR "stroke"[MeSH Terms]    Proquest search terms:  noft(stroke* OR "cerebrovascular accident" OR "post-stroke" OR "stroke survivor*" OR "stroke"[MeSH Terms] )  Web of Science, Scopus search terms:  TOPIC: stroke* OR "cerebrovascular accident" OR "post-stroke" OR "stroke survivor*" OR "stroke"[MeSH Terms]  ScienceDirect search terms:  stroke[tiab] OR strokes[tiab] OR "cerebrovascular accident"[tiab] OR "post-stroke"[tiab] OR "stroke survivor"[tiab] OR OR "stroke survivors"[tiab] OR "stroke"[MeSH Terms] | 219,788 | 367,387 | 321 | 311,764 | 479,728 |
| **Concept 2: Online community**  PubMed search terms:  "online community"[tiab] OR "virtual community"[tiab] OR "support group*"[tiab] OR "social media"[tiab] OR "online forum*"[tiab] OR "peer support"[tiab] OR caregiver*[tiab] OR "social support"[MeSH Terms] OR "Self-Help Groups"[Mesh] OR "Social Environment"[Mesh] OR "Caregivers"[Mesh]  Proquest search terms:  noft("online community" OR "virtual community" OR "support group*" OR "social media" OR "online forum*" OR "peer support" OR caregiver* OR "social support"[MeSH] OR "Self-Help Groups"[Mesh] OR "Social Environment"[Mesh] OR "Caregivers"[Mesh])  Web of Science, Scopus search terms:  "online community" OR "virtual community" OR "support group*" OR "social media" OR "online forum*" OR "peer support" OR caregiver* OR "social support"[MeSH] OR "Self-Help Groups"[Mesh] OR "Social Environment"[Mesh] OR "Caregivers"[Mesh] (Topic)  ScienceDirect search terms:  "online community"[tiab] OR "virtual community"[tiab] OR "support group"[tiab] OR "support groups"[tiab] OR "social media"[tiab] OR "online forum"[tiab] OR "online forumd"[tiab] OR "peer support"[tiab] caregiver[tiab] OR caregivers[tiab] OR "social support"[MeSH Terms] OR "Self-Help Groups"[Mesh] OR "Social Environment"[Mesh] OR "Caregivers"[Mesh] | 166,526 | 1,011,519 | 597 | 269,895 | 795,319 |
| **Concept 3: Digital technology**  Pubmed search terms:  technolog*[tiab] OR digital[tiab] OR online[tiab] OR virtual[tiab] OR "web-based"[tiab] OR eHealth[tiab] OR mHealth[tiab] OR telehealth*[tiab] OR telerehab*[tiab] OR app[tiab] OR apps[tiab] OR "Software"[Mesh] OR "Internet-Based Intervention"[Mesh] OR "Virtual Reality"[Mesh]  Proquest search terms:  noft(technolog* OR digital OR online OR virtual OR "web-based" OR eHealth OR mHealth OR telehealth* OR telerehab* OR app OR apps OR "Software"[Mesh] OR "Internet-Based Intervention"[Mesh] OR "Virtual Reality"[Mesh])  Web of Science, Scopus search terms:  technolog* OR digital OR online OR virtual OR "web-based" OR eHealth OR mHealth OR telehealth* OR telerehab* OR app OR apps OR "Software"[Mesh] OR "Internet-Based Intervention"[Mesh] OR "Virtual Reality"[Mesh]  ScienceDirect search terms:  technology[tiab] OR technologies[tiab] OR technological[tiab] OR digital[tiab] OR online[tiab] OR virtual[tiab] OR "web-based"[tiab] OR eHealth[tiab] OR mHealth[tiab] OR telehealth[tiab] OR telerehab[tiab] OR telerehabilitation[tiab] OR app[tiab] OR apps[tiab] OR "Software"[Mesh] OR "Internet-Based Intervention"[Mesh] OR "Virtual Reality"[Mesh] | 1,032,230 | 129,976,043 | 31,563 | 3,353,728 | 1,660,981 |
| Combined search: 1 [AND] 2 [AND] 3 | 493 | 1,208  Filtered to scholarly journals only:  630 | 5 | 759 | 661 |
| **Notes**  Used ‘Social Environment’ [Mesh], which is a higher order concept in the Mesh categorisation.  Used ‘Software’ [Mesh] rather than ‘Mobile Applications’ [Mesh], again because it is a higher order concept. |  | Exclude: Wire Feeds, Newspapers, Blogs/Podcasts/Websites, Trade Journals, Other Sources, Conference Papers, Dissertations/  Theses, Magazines, Working Papers/Reports, Commentary |  |  | Wildcards (*) not permitted in ScienceDirect |
