# Supplementary material for: Online Community Support for Stroke Survivors and Caregivers: Scoping Review
Source: J Med Internet Res. 2026 Apr 29;28:e71190. doi: 10.2196/71190 (PMC13127857; doi:10.2196/71190)
Supplement: Multimedia Appendix 2 [file jmir-v28-e71190-s002.docx]

**Data Chart Item**

| **Authors** | **Title** | **Topic** | **Journal** | **Country** | **Aim** | **Methodology** | **Respondent** | **Result** |
| --- | --- | --- | --- | --- | --- | --- | --- | --- |
| Gooch et al. [1] | Behavior Change Approaches in Digital Technology–Based Physical Rehabilitation Interventions Following Stroke: Scoping Review | Community functions and roles | Journal of Medical Internet Research | World-  wide | To identify if and how behavior change approaches (i.e., theories, models, frameworks, and techniques to influence behavior) are incorporated within physical stroke rehabilitation interventions that include a digital health technologies (DHT) | Review | Stroke survivors,  Stroke caregivers,  Health workers | Virtual reality was the most frequently identified DHT type (56%). Almost two-thirds (63%) of studies focused on upper limb rehabilitation. Only a limited number of studies (17%) included a theory, model, or framework for behavior change. The most frequently used behavior change technique taxonomy clusters were feedback and monitoring (85%); reward and threat (54%); goals and planning (32%); and shaping knowledge (32%). A relationship map of feedback and monitoring with reward and threat indicated prominent use of interventions that included virtual reality.  This review challenged assumptions that DHTs promote engagement in rehabilitation, by demonstrating that very few studies of DHT in physical stroke rehabilitation overtly used any behavior change approach. |
| Reszel et al. [2] | How Community-Based Teams Use the Stroke Recovery in Motion Implementation Planner: Longitudinal Qualitative Field Test Study | Online stroke community system | JMIR Formative Research | Canada | To describe how teams used the Planner in real-world conditions, explore the effects of the Planner on participants' knowledge, attitudes, and activities related to implementation planning, and identify factors that influenced the usage of the Planner. | Longitudinal Qualitative design | Health workers | The Planner was helpful in building capacity in health worker teams. It was used in diverse, non-linear ways, and was adapted to local contexts. The Planner positively impacted teams' planning capabilities by: increasing knowledge about the recovery planning process, improving attitudes toward community engagement, and influencing planning activities. The study also identified ongoing challenges related to organizational context, leadership, and the COVID-19 pandemic in Planner usage. |
| Pindus et al. [3] | Stroke survivors’ and informal caregivers’  experiences of primary care and community  healthcare services – A systematic review and  meta-ethnography | Community functions and roles | PLOSONE | World-wide | To describe and explain stroke survivors and informal caregivers’ experiences of primary care and community healthcare services.  To offer potential solutions for how negative experiences could be addressed by healthcare services | Review | Stroke survivors,  Stroke caregivers | From 51 studies (including 168 stroke survivors and 328 caregivers), three inter-dependent third order constructs were developed: (1) marginalisation of stroke survivors and caregivers by healthcare services, (2) passivity versus proactivity in the relationship between health services and the patient/caregiver dyad, and (3) fluidity of stroke-related needs for both patient and caregiver. These were driven by issues of continuity of care, limitations in access to services and inadequate information provision. The need to consider changing information needs and psychological adaptation in the long-term following stroke was highlighted. |
| White et al. [4] | Predictors of health-related quality of life in  community-dwelling stroke survivors: a cohort  study | Community functions and roles | Health Service Research | Australia | To assess change in levels of, and identify independent predictors of, health-related quality of life (HRQoL) over the first 12-months post-stroke | Prospective Cohort study | Stroke survivors | Impaired HRQoL did not change significantly with time post-stroke. Higher HRQoL scores were independently associated with higher baseline HRQoL (P = 0.03), younger age (P = 0.006), lower disability (P = 0.003), greater community participation (P ≤ 0.001) and no history of depression (P = 0.03). |
| Feigin et al. [5] | Pragmatic solutions to reduce the global burden of stroke:  a World Stroke Organization | Stroke ecosystem | Lancet Neurology Commissions | World-wide | To forecast the burden of stroke from 2020 to 2050, to identify the barriers to, and facilitators for the achievement of the four pillars. | Review | Members of the World Stroke Organization-Lancet Neurology Commission Stroke Collaboration Group (including Consumer representatives, health workers,  researchers, funders,  policy makers) | Pragmatic solutions were proposed for implementing evidence-based interventions across each of the four pillars developed to reduce the global burden of stroke: surveillance, prevention, acute care, and rehabilitation. Pragmatic solutions should help to mitigate the  estimated direct (i.e., treatment and rehabilitation) and indirect (considering productivity loss) costs of stroke globally (in excess of US$891 billion annually). Such solutions will also aid in reducing the global burden of stroke, contribute to achievement of SDG 3.4 (reduce premature death from non-communicable diseases) and key World Health Organization (WHO) Global Action Plans on epilepsy and other neurological disorders (2022–2031) and for the prevention and control of non-communicable diseases (2013-2030). |
| Fu et al. [6] | Effects of A Benefit-Finding Intervention in Stroke Caregivers in Communities | Community functions and roles | Clinical Gerontologist | China | To verify the effectiveness and feasibility of a nine-week benefit-finding intervention on reducing caregiver burden, enhancing caregiver benefit finding, and improving the quality of life of both caregivers and stroke survivors. | RCT | Stroke survivors,  Stroke caregivers | The intervention significantly improved caregiver benefit-finding, reduced caregiver burden, and enhanced quality of life for caregivers and stroke survivors compared to the control group. Study results indicate that the benefit-finding intervention is feasible and effective in improving the health outcomes for caregivers and stroke survivors​. |
| Cameron & Gignac [7] | "Timing It Right": A conceptual framework for addressing the support needs of family caregivers to stroke survivors from the hospital to the home | Community functions and roles | Patient Education and Counseling | Canada | Provide a framework that describes how caregivers' support needs change throughout different phases of the care continuum for stroke survivors, from the acute hospital setting to the home, in order to guide healthcare professionals in offering timely and appropriate support, education, and training to caregivers. | Review | Stroke caregivers | Development of the "Timing It Right" framework identified gaps in caregiver education, training, and support.The framework identifies five distinct phases of caregiving: (1) Event/diagnosis, (2) Stabilization, (3) Preparation, (4) Implementation, (5) Adaptation. The framework identifies the phase-specific characteristics in terms of care needs, caregiver responsibilities, and support required.  Addressing these offers the potential to enhance caregiver well-being and rehabilitation outcomes of stroke survivors. |
| Lobo et al. [8] | Caregiver Engagement in Stroke Care: Opportunities and Challenges in Australia and Denmark. | Community functions and roles | Frontiers in Public Health | Australia, Denmark | To understand the needs of the caregiver in stroke that limit engagement, and processes employed in countries such as Australia and Denmark to provide care for the person with stroke. | Review | Stroke survivors,  stroke caregivers,  health workers | Australia and Denmark have similar foci of community-based stroke care, which necessitates greater understanding of the factors that shape caregiver engagement. The study emphasises the need to develop caregiver support strategies that are targeted to different levels of care need and stages of stroke recovery. This has the potential to reduce unmet needs and promote ongoing interaction with health professionals on an on-going basis, but relies upon building relationships between various stakeholders. Study findings emphasise the need to include public health policies that promote caregiver engagement; especially given the community focus of stroke care. |
| Leonardi & Fheodoroff [9] | Goal setting with ICF (International Classification of Functioning, Disability and Health) and multidisciplinary team approach in stroke rehabilitation | Community functions and roles | Clinical Pathways in Stroke Rehabilitation | World-wide | Highlight the importance of goal setting using the ICF framework in stroke rehabilitation and emphasize the value of an interdisciplinary approach. The goal is to improve the quality of care, optimize rehabilitation outcomes, and facilitate the reintegration of stroke patients into their communities. | Review | Health workers | The ICF framework provides a comprehensive, holistic view for health professionals to understand stroke survivors’ functioning and needs in order to set recovery goals that are realistic, measurable, and adaptable over time. The study concludes that this approach helps reduce care fragmentation, enhances communication across different health professionals, and leads to more effective rehabilitation planning and outcomes. |
| Aravind et al. [10] | Community-based exercise programs  incorporating healthcare-community  partnerships to improve function post-stroke:  feasibility of a 2-group randomized controlled  trial | Community functions and roles | Pilot and Feasibility Studies | Canada | To evaluate the feasibility and acceptability of a community-based exercise programs  incorporating healthcare-community  partnerships (CBEP-HCP) progam compared to a waitlist control group to improve everyday function among people post-stroke. | RCT | Stroke survivors,  Stroke caregivers | Thirty-three people with stroke were randomized to the intervention (n = 16) or waitlist (control) group (n = 17).  Five intervention group participants (31%) attended fewer than 50% of classes for health reasons, and retention in the intervention and waitlist groups was 88% and 82%, respectively. Attendance at 10-month evaluations was 63% and 71%, respectively. Barriers to participation in exercise classes and evaluation included inclement weather, transportation availability, and commute duration. Among those who attended ≥ 50% of classes, results suggested an immediate positive effect on balance, balance self-efficacy, lower limb strength, everyday function, and overall health.  Methodological insights include participants’ preference to being recruited by a familiar healthcare professional, and that a 10- or 12-month waitlist duration was too long. |
| Smith et al. [11] | “I Cannot Do All of This Alone”: Exploring Instrumental and  Prayer Support in Online Health Communities | Online stroke community system | ACM Trans. Computer-Human Interactions | World-wide | Through partnership with an online health community (OHC), aimed to understand what OHC users acknowledge and prioritise in terms of social support as part of living with illness; to understand the types of instrumental support that are wanted or preferred. | Mixed methods | Stroke survivors,  Stroke caregivers | Patients and family caregivers prefer to receive different types of support than their broader care networks generally provide. In addition, people generally have more trust in their closest social connections than acquaintances or businesses to provide instrumental support. Users rated “prayer support” as the most important support category to them, followed by emotional or instrumental support. Informational support was not mentioned. Findings indicate the need to accommodate divergent preferences and to expand instrumental support networks in OHC design to empower family caregivers and support spirituality. |
| Magwood et al. [12] | Community-based interventions for stroke provided by nurses and community health workers: A review of the literature | Community functions and roles | Journal of Neuroscience Nursing | World-wide | To examine studies that target enhancing community-based stroke recovery for adult stroke survivors, caregivers, or both, through interventions provided by nurses or community health workers (CHWs). | Review | Stroke survivors,  Stroke caregivers,  Health workers | Insights from eighteen studies identified that nurses and CHWs are crucial in community-based stroke recovery, prevention of recurrent stroke, and play an integral role in supporting caregivers. More comprehensive programs are needed to address the complex needs of stroke survivors and families returning home. Such interventions provide improvements in health perception, quality of life, knowledge, self-efficacy, and caregiver support. |
| Wright et al. [13] | Exploring the Types of Social Support Exchanged by Survivors of Pediatric Stroke and Their Families in an Online Peer Support Community: Qualitative Thematic Analysis | Community functions and roles | Journal of Medical Internet Research | United Kingdom | To identify if social support was exchanged within an online community for pediatric stroke survivors and their families, and to analyze the type of support provided. | Thematic content analysis | Stroke survivors,  Stroke caregivers,  Community/ society | Pediatric stroke survivors and their families actively engaged in the online community, exchanging various types of social support, with informational and emotional support being the most common. Long-term survivors of pediatric stroke were particularly important in providing informational support from their lived experiences. The study highlighted the potential of online communities to address unmet rehabilitation needs, though more research is required to assess health and rehabilitation outcomes from such engagement. |
| Millar et al. [14] | What is important in supporting self-management in community stroke rehabilitation? A Q methodology study | Community functions and roles | Disability and Rehabilitation | United Kingdom | Explore the key factors that contribute to the effectiveness of supported self-management (SSM) in stroke rehabilitation within the community. | Mixed methods | Stroke survivors,  Health workers | Four distinct viewpoints regarding what makes SSM work were identified: (1) A person-centered approach to build self-confidence and self-worth; (2) Feeling heard, understood, and supported by everyone; (3) Proper preparation, appropriate resources, and training; and (4) Ensuring the right support is provided at the right time for the individual.  All viewpoints emphasized the importance of a trusting, supportive relationship between stroke survivors and clinicians, meaningful goal setting, and building self-worth and confidence as core components of SSM. |
| Deutschbein et al. [15] | Community care coordination for stroke survivors: results of a complex intervention study | Community functions and roles | BMC Health Services Research | Germany | To assess the effectiveness of a care coordination program for stroke survivors, and to determine if the program improves health service utilization, reduces hospital readmissions, lowers costs, and impacts recurrent stroke events and mortality rates. | Pragmatic non-randomized intervention trial | Stroke survivors | The care coordination program led to increased utilization of outpatient neurologist services and significantly reduced hospital readmissions and related costs. However, there was no significant effect on recurrent stroke rates or mortality. Care coordination improves the overall organization and use of health services for stroke survivors, potentially preventing unnecessary hospitalizations and improving cost efficiency. |
| Norlander et al. [16] | Participation in social and leisure activities while re-constructing the self: understanding strategies used by stroke survivors from a long-term perspective | Community functions and roles | Disability and Rehabilitation | Sweden | To gain a better understanding of social and leisure (SL) participation post-stroke and how it can be supported over the long term, this study seeks to explore the strategies employed by long-term stroke survivors in relation to their SL activities. | Grounded Theory | Stroke survivors,  Stroke caregivers | Nine strategies were used by participants for two main purposes: to protect and re-construct their post-stroke identity and to enable participation in meaningful social and leisure (SL) activities despite the challenges. The strategies for SL participation involve balancing various life priorities and evolve over time, influenced by personal and environmental factors. These insights enhance the understanding of why and how stroke survivors maintain, abandon, or adopt new activities and underscore the importance of adopting a long-term perspective in stroke rehabilitation and support. |
| Thomas et al. [17] | How is poststroke fatigue understood by stroke survivors and carers? A thematic analysis of an online discussion forum | Online stroke community system | BMJ Open | United Kingdom | To explore how stroke survivors and their caregivers perceive and describe poststroke fatigue in an online discussion forum. | Thematic content analysis | Stroke survivors,  Stroke caregivers | Six key themes on how stroke survivors and caregivers perceive poststroke fatigue (PSF) were identified: (1) Medicalization: Fatigue is seen as a typical stroke symptom; (2) Unique to Stroke: Described as a distinct, long-lasting tiredness; (3) Acceptance: Survivors normalize fatigue as part of stroke recovery; (4) Fighting Fatigue: Some try to actively resist it; (5) Biological Explanations: Fatigue is often linked to brain healing; and (6) Coping Mechanisms: Strategies include pacing oneself and adapting to new limitations.  Findings emphasize the need for better understanding and communication from healthcare professionals regarding PSF. |
| Scrivener et al. [18] | Feasibility of a Self-Managed, Video-Guided Exercise Program for Community-Dwelling People with Stroke | Digital support technologies | Stroke Research and Treatment | Australia | Determine the feasibility of delivering the TASK (a video-guided exercise program) with a self-management approach; evaluate key aspects of feasibility such as safety, adherence, acceptability, and cost; and assess the impact of the program on physical activity, walking ability, and exercise self-efficacy. | Phase I, single-group clinical trial | Stroke survivors | The program’s feasibility was supported, with insights related to five themes: (1) Adherence: Participants adhered well to the program, with higher adherence in the supervised phase than in the self-directed phase; (2) Safety: No adverse events were reported, indicating the program can be implemented safely; (3) Acceptability: Most participants (92%) found the program easy to use and 86% would recommend it to others; (4) Cost: The program was estimated to cost $737 AUD per participant for 8 weeks, including equipment and therapist time; and (5) Outcomes: While there were improvements in walking speed, there were no significant changes in self-efficacy or physical activity over the duration of the program. |
| Olafsdottir et al. [19] | Developing ActivABLES for community-dwelling stroke survivors using the Medical Research Council framework for complex interventions | Digital support technologies | BMC Health Services Research | Finland, Iceland, Sweden | Develop ActivABLES, a set of tools and applications to support stroke survivors in performing home-based exercises and physical activities, enhancing their rehabilitation process and quality of life. | Multi-phase, mixed methods intervention study | Stroke survivors,  Stroke caregivers,  Health workers | The development of ActivABLES was successful in creating a set of tools to promote home-based exercises for stroke survivors. Stroke survivors and caregivers found the tools engaging and motivational, emphasizing the importance of visual and auditory feedback for exercise adherence. Six relevant prototypes for home-based rehabilitation were developed:  (1) ActivFOAM: For balance exercises, with pressure sensors and interactive games for weight-shifting tasks.  (2) WalkingSTARR: A step-counting app designed to encourage walking.  (3) ActivBALL: A ball for hand and arm exercises, providing tailored instructions and feedback.  (4) ActivSTICKS: Tools for upper arm exercises involving interactive movements.  (5) ActivLAMP and (6) ActivTREE: Devices providing visual feedback on daily exercise progress, motivating users by lighting up as exercise goals are met. |
| Lee et al. [20] | Comparison of individualized virtual reality- and group-based rehabilitation in older adults with chronic stroke in community settings: a pilot randomized controlled trial | Digital support technologies | European Journal of Integrative Medicine | South Korea | To evaluate and compare the effects of individualized VR-based and group-based rehabilitation on upper extremity motor function, activities of daily living (ADL) performance, and health-related quality of life (HRQoL) in community-dwelling older adults with chronic stroke. The study also seeks to assess the acceptability of both rehabilitation methods, including attendance rate, satisfaction, and intention of adherence. | RCT | Stroke survivors | Findings indicate that VR-based rehabilitation may be more effective in improving objectively measured upper extremity function, especially in the proximal part of the arm. Group-based rehabilitation may be more beneficial for improving self-reported ADL performance and HRQoL. Attendance rates were slightly higher in the VR-based group (95.1%) compared to the group-based rehabilitation group (88.6%). Both groups reported high levels of satisfaction and intention to adhere to the rehabilitation programs. |
| Olafsdottir et al. [21] | Feasibility of ActivABLES to promote home-based exercise and physical activity of community-dwelling stroke survivors with support from caregivers: A mixed methods study | Digital support technologies | BMC Health Services Research | Finland, Iceland, Sweden | To evaluate the feasibility of using ActivABLES to promote home-based exercise and physical activity among community-dwelling stroke survivors with support from their caregivers. It includes the acceptability, demand, implementation, and practicality of the ActivABLES intervention. | Convergent mixed-methods design | Stroke survivors,  stroke caregivers | Functional improvements were noted in balance and mobility, with increases in Berg Balance Scale scores (+2.5), Activities-Specific Balance Confidence Scale (+0.9), improvements in Timed-Up-and-Go (-4.2 seconds), and Five Times Sit to Stand Test (-2.7 seconds).  Physical activity increased, as indicated by motion detectors (e.g., more steps, increased standing time).  Interviews identified themes for each feasibility domain:  (1) Acceptability: Stroke survivors and caregivers expressed appreciation for the ActivABLES tools. Functional improvements and increased self-initiated activities were reported; (2) Demand: Most participants showed interest in further use, with high engagement during the four-week period; (3) Implementation: Feedback mechanisms and exercise progression were considered important. Some issues related to technical challenges were reported; and (4) Practicality: The need for technical support was noted, but caregivers were generally able to provide assistance. |
| Camicia et al. [22] | Nursing's Role in Successful Stroke Care Transitions Across the Continuum: From Acute Care Into the Community | Digital support technologies | Stroke | World-wide | To provide evidence on the critical roles that nurses play in managing stroke care transitions across various healthcare settings. To address cross-setting issues in stroke care.  To provide recommendations for enhancing nursing’s impact in improving outcomes for stroke survivors and their families across the entire care continuum​. | Review | Stroke survivors,  stroke caregivers,  health workers (nurses) | Key recommendations to improve nursing's role in stroke care transitions include:  (1) Establishing coordinated and seamless comprehensive stroke care across the continuum into the community.  (2) Implementing a stroke nurse liaison role for better case management and communication across all settings.  (3) Using validated tools like the PATH-s to assess caregiver preparedness.  (4) Employing evidence-based teaching methods to improve patient and caregiver education.  (5) Leveraging technology to advance nursing care and optimize outcomes for stroke survivors​.  These measures are aimed at addressing inefficiencies in post-acute care transitions and improving the overall health and quality of life for stroke survivors and their caregivers. |
| Demers et al. [23] | Wearable Technology to Capture Arm Use of People With Stroke in Home and Community Settings: Feasibility and Early Insights on Motor Performance | Digital support technologies | Physical Therapy & Rehabilitation Journal | United States of America | To establish the short-term feasibility and usability of wrist-worn wearable sensors for capturing the arm and hand activity of people with stroke and to explore the association between factors related to the use of the paretic arm and hand. | Quantitative multi-phased observational study | Stroke survivors | Valid data was collected on 87.6% of days, with an average wear time of 12.6 hours per day. Minimal technical issues were reported, though a few participants experienced discomfort or unrelated adverse events. Usability scores were high, with a mean score of 85.4/100. Significant correlations were found between motor capacity, self-efficacy, and arm use in real-life settings. The study highlights the potential of wearable technology to enhance clinical practice by providing insights into arm use outside of therapy. |
| Freund et al. [24] | Effectiveness of information and communications technology interventions for stroke survivors and their support people: a systematic review | Digital support technologies | Disability and Rehabilitation | World-wide | To examine the effectiveness of self-directed, off-the-shelf information and communications technology (ICT)-based interventions in improving the quality of life, physical and psychosocial outcomes of community-dwelling stroke survivors and their support persons (SP). | Review | Stroke survivors,  stroke caregivers | ICT-based interventions with minimal clinician supervision can provide benefits to stroke survivors and their SPs. Fifteen studies reported significant positive effects for at least one outcome, including stroke-specific, physical, behavioral, and health service use outcomes. However, none of the studies found significant improvements in psychosocial well-being. Adherence to interventions was variable, but the few studies that reported on intervention acceptability generally found good acceptability. |
| Johnson et al. [25] | An Innovative STRoke Interactive Virtual thErapy (STRIVE) Online Platform for Community-Dwelling Stroke Survivors: A Randomized Controlled Trial | Digital support technologies | Archives of Physical Medicine and Rehabilitation | Australia | To investigate the STRoke Interactive Virtual thErapy (STRIVE) intervention on upper-extremity clinical outcomes in community- dwelling stroke survivors. | RCT | Stroke survivors | Participants in the virtual therapy (VT) group experienced significant improvements in motor function of the upper extremity, as measured by the Fugl-Meyer Upper Extremity (FMUE) scale, indicating that the virtual therapy contributed to meaningful motor function gains.  No significant changes were noted in the Action Research Arm Test (ARAT) scores.  Spasticity and Motor Use: The VT group showed reduced spasticity in the shoulder and elbow, and there were increases in daily arm use and quality of movement.  Safety: No adverse events were reported, demonstrating that the program was safe and well-tolerated. |
| Berkeley et al. [26] | Referral to Community-Based Rehabilitation Following Acute Stroke: Findings From the COMPASS Pragmatic Trial | Community functions and roles | Circulation: Cardiovascular Quality and Outcomes | United States of America | To evaluate the extent to which stroke survivors discharged home were referred to community-based rehabilitation and to identify patient, hospital, and community-level predictors of referral.  To understand the referral rates for physical, occupational, and speech therapy at discharge following stroke or transient ischemic attack. | Secondary observational analysis of a cluster-randomized trial | Stroke survivors | Approximately one-third (36%) of stroke survivors were referred to community-based rehabilitation, with notable variability across hospitals. Considerable variation in referral rates across hospitals could not be fully explained by the measured factors, suggesting opportunities for improved poststroke rehabilitative care.  Rates of referral to physical, occupational, and speech therapists were 31%, 18%, and 10%, respectively. Referral rates by hospital ranged from 3% to 78% with a median of 35%. Patient-level factors, such as higher stroke severity, medical comorbidities, older age, female sex, non-White race, and having Medicare insurance were significant predictors of referral. Referral was higher for patients living in counties with greater physical therapist supply. |
| Juengst et al. [27] | Use of mHealth Technology for Patient-Reported Outcomes in Community-Dwelling Adults with Acquired Brain Injuries: A Scoping Review | Digital support technologies | International Journal of Environmental Research and Public Health | World-wide | To describe the current use of mHealth technology for long-term assessment of patient-reported outcomes in community-dwelling individuals with acquired brain injury (ABI). | Review | Stroke survivors | Collecting patient-reported outcomes via mHealth technology is both feasible and acceptable in individuals with chronic ABI. The review highlights the advantages of using Ecological Momentary Assessment (EMA) over single time-point assessments, especially for fluctuating symptoms such as mood, fatigue, and affect. MHealth technologies allow for more consistent and timely data collection, which can better capture variability in symptoms and potentially support behavior change. |
| Kerr et al. [28] | Adoption of Stroke Rehabilitation Technologies by the User Community: Qualitative Study | Digital support technologies | JMIR Rehabilitation and Assistive Technologies | United Kingdom | To identify stakeholder (therapists, patients, and caregivers) priorities for stroke rehabilitation technologies and to generate user-centered solutions for enhancing everyday adoption. | Consensus building methods | Stroke survivors,  stroke caregivers,  health workers,  community/  society,  policy makers, tech developers | 25 priorities were generated, reduced to 10 through discussion and voting at Workshop 1: access to technologies, ease of use, awareness, functionality, self-management support, user training, effectiveness, cost value, knowledgeable staff, and performance feedback. Workshop 2 recommended strategies to improve technology adoption in stroke rehabilitation, including an annual technology exhibition, a consumer-rating website, and a user network to test and inspire new technologies. The workshops highlighted the importance of collaboration between developers and users to promote evidence-based stroke rehabilitation and drive technology adoption. |
| Krishnan et al. [29] | Perceptions of stroke survivors regarding factors affecting adoption of technology and exergames for rehabilitation | Digital support technologies | PM&R (Physical Medicine and Rehabilitation) | United States of America | To investigate perceptions of individuals with stroke regarding technology and exergames for rehabilitation. | Phenomenology | Stroke survivors | Individuals with stroke had limited experiences using exergames and VR/AR devices. They were motivated to use technologies and exergames to promote functional recovery. Participants identified facilitators (e.g., enhancing functional recovery, feedback, therapist supervision) and barriers (e.g., safety, inaccessibility, inadequate knowledge) to adopting exergames in their daily lives. Participants wanted the exergames to be customizable, goal oriented, and enjoyable to maintain their engagement. However, they indicated that these games could not replace the therapist’s supervision. |
| Lam et al. [30] | Evaluation of Cost-Effectiveness of a Virtual Multidisciplinary Stroke Care Clinic for Community-Dwelling Survivors of Stroke | Digital support technologies | Journal of the American Heart Association | Hong Kong | To evaluate the cost-effectiveness of a Virtual Multidisciplinary Stroke Care Clinic (VMSCC) service. This service integrated technology with multidisciplinary stroke care and included virtual consultations with a nurse, home blood pressure telemonitoring, and access to an online resource platform, alongside usual care. | RCT | Stroke survivors | The VMSCC service resulted in greater reductions in emergency admissions and days of hospitalization compared to usual care, but it incurred higher total costs. The service led to an average reduction of 0.06 emergency admissions and 0.08 days of hospitalization, with an additional cost of HK$375 (~US$48). The incremental cost-effectiveness ratios were HK$6070 and HK$4826 for each emergency admission and day of hospital stay reduced, respectively. |
| Lobo et al. [31] | Understanding the Methodological Issues and Solutions in the Research Design of Stroke Caregiving Technology | Digital support technologies | Frontiers in Public Health | World-wide | To address the challenges related to the design and implementation of health technologies for stroke caregiving.  To understand the potential of health technologies to support stroke caregivers and identify the methodological issues in designing such technologies. | Review | Stroke caregivers | Significant methodological issues that hinder the effective design and adoption of stroke caregiving technologies were identified, including: (1) a lack of user-centered approaches, (2) limited understanding of caregivers' needs, and (3) challenges in the technical and organizational implementation of such technologies. The study recommends user-centered design practices, emphasizing the involvement of caregivers in the design process, and in suggesting strategies to better tailor technological solutions to meet their needs​. |
| Lo et al. [32] | Virtual Multidisciplinary Stroke Care Clinic for Community-Dwelling Stroke Survivors: A Randomized Controlled Trial | Digital support technologies | Stroke | Hong Kong | To examine the effects of a virtual multidisciplinary stroke care clinic on the health and self-management outcomes of stroke survivors. | RCT | Stroke survivors | Participants in the intervention group had significantly greater improvements in self-efficacy, social participation, and depression, but no significant improvement in self-management behaviors compared to the control group. The effect sizes were small to medium, which may not be of clinical relevance. |
| Luo et al. [33] | Development of a compensation-aware virtual rehabilitation system for upper extremity rehabilitation in community-dwelling older adults with stroke | Digital support technologies | Journal of NeuroEngineering and Rehabilitation | Singapore | To develop a compensation‐aware virtual rehabilitation system (VRS) that can detect compensatory movements and improve the outcome of upper extremity rehabilitation in community‐dwelling older adults with stroke. | Intervention study | Stroke survivors,  Community/  society | The VRS could effectively detect all determined compensatory movements and timely trigger feedback in response to the detected compensatory movements. The virtual rehabilitation participants showed significant improvements in Fugl-Meyer Assessment‐Upper Extremity (FMA‐UE, p = 0.045) and Wolf Motor Function Test ( WMFT, p = 0.009). However, the VR and conventional training groups had no significant differences in outcome measures. |
| Murakami et al. [34] | Impaired Higher-Level Functional Capacity as a Predictor of Stroke in Community-Dwelling Older Adults: The Ohasama Study | Community functions and roles | Stroke | Japan | To investigate whether impaired higher-level functional capacity is a predictor of the first incidence of stroke in older adults living independently in the community. | Longitudinal observational study | Stroke survivors,  Community/  society | Impaired higher-level functional capacity, particularly in intellectual activity, was a significant predictor of stroke. The hazard ratio for stroke associated with impaired intellectual activity was 1.64, and for those aged 75 and older, impaired social role was also significantly associated with stroke. This suggests that monitoring higher-level functional capacity, especially intellectual activities, could help identify individuals at higher risk of stroke​. |
| Peters et al. [35] | Utilization of wearable technology to assess gait and mobility post-stroke: a systematic review. | Digital support technologies | Journal of NeuroEngineering and Rehabilitation | World-wide | To identify how wearable technologies have been used over the past decade to assess gait and mobility in persons with stroke. | Review | Stroke survivors | The use of various wearable technologies has enabled researchers and clinicians to monitor patients’ activity in a multitude of settings post-stroke. Using data from wearables may provide clinicians with insights into their patients’ lived-experiences and enrich their evaluations and plans of care. Accelerometers, activity monitors, and pressure sensors were the most commonly used wearable technologies to assess gait and mobility post-stroke. The most widely assessed spatiotemporal parameters of gait were gait speed and cadence, and the most common mobility measures included step count and duration of activity. |
| Sarfo et al. [36] | Knowledge and perspectives of community members on risk assessment for stroke prevention using mobile health approaches in Nigeria | Digital support technologies | Journal of Stroke and Cerebrovascular Diseases | Nigeria | To assess the knowledge of community dwelling adults on stroke risk and their willingness to use mobile health (mHealth) technology in assessing their stroke risk. | Cross-sectional observational study | Community members | 84% of participants wanted to know their risk for developing stroke, but only 29.6% of respondents previously had their stroke risk assessed. Factors associated with willingness to know stroke risk were age and ethnicity. 66% of participants wanted to know their immediate risk of stroke, compared with. 6.6% and 2.1% who wanted to know their 5- or 10-year future stroke risks. Regarding locations, participants preferred stroke risk assessment to be performed at a health facility, at home by health professional, on their own using mHealth (stroke risk calculator application), or at communal gatherings. About 70% wished to learn about their stroke risk via mHealth application. |
| Saywell et al. [37] | Telerehabilitation After Stroke Using Readily Available Technology: A Randomized Controlled Trial | Digital support technologies | Neurorehabilitation and Neural Repair | New Zealand | To investigate whether the Augmented Community Telerehabilitation Intervention (ACTIV), a 6-month program involving a combination of face-to-face sessions, telephone contact, and text message reminders, could improve physical function for people with stroke. | RCT | Stroke survivors | ACTIV did not significantly improve physical function in the intervention group compared to the control group based on the intention-to-treat analysis. However, in the per-protocol analysis, which included participants who received at least 50% of the intervention, there was a significant improvement in physical function. Despite these improvements, gains were not maintained at the 12-month follow-up. |
| Silvera-Tawil et al. [38] | Multicomponent Support Program for Secondary Prevention of Stroke Using Digital Health Technology: Co-Design Study With People Living With Stroke or Transient Ischemic Attack | Digital support technologies | Journal of Medical Internet Research | Australia | To co-design a multicomponent digital technology support program for the secondary prevention of stroke, with input from both people with lived experience of stroke or transient ischemic attack (TIA) (referred to as consumers) and clinicians. | Co-design study | Stroke survivors,  health workers | Most clinicians indicated the importance of monitoring health and lifestyle measures more frequently than current practice, particularly physical activity, weight, and sleep. Most consumers and clinicians agreed that providing alerts about potential deterioration in an individual’s condition were important functions. Intention to use a digital program for stroke prevention was high. Participants endorsed using a digital health program to help manage stroke or TIA and discussed preferred functions and health measures in a digital solution for secondary prevention of stroke. Clinician feedback highlighted the need for a customizable clinician portal that captures individual consumer goals. The result of the study is the development of a consumer-facing app that integrates wearable activity trackers and a customizable clinician web portal. The app was designed to help people manage their health post-stroke, with functions such as monitoring health and lifestyle measures, providing alerts about potential deterioration, and supporting communication with clinicians. |
| Sun et al. [39] | A Survey of Technologies Facilitating Home and Community-Based Stroke Rehabilitation | Digital support technologies | International Journal of Human–Computer Interaction | World-wide | To provide a comprehensive survey of the current status of technologies facilitating home-based, community-based, emphasizing the importance of remote rehabilitation techniques that allow therapist-patient collaboration. | Review | Stroke survivors,  Stroke caregivers,  Health workers,  Community/  society | The study provides a detailed review of existing technologies and applications for physical, speech, and assessment rehabilitation. It highlights the various technologies supporting remote therapist-patient interactions in stroke rehabilitation. Critical issues that need further exploration are identified, and potential future research areas to enhance the effectiveness of home and community-based rehabilitation strategies​ are proposed. |
| Xu et al. [40] | Developing a falls prevention program for community-dwelling stroke survivors in Singapore: client and caregiver perspectives | Digital support technologies | Disability and Rehabilitation | Singapore | To explore the experiences of stroke survivors, family caregivers, and domestic helpers regarding self-perceived fall risk factors, common fall prevention strategies used, and challenges to community participation after a fall. | Thematic analysis | Stroke survivors,  stroke caregivers | Stroke survivors and caregivers face both intrinsic and extrinsic risk factors for falls. Falls lead to significant challenges such as fear of falling, increased self-care needs, and restricted mobility and social participation. Caregivers prioritize safety, often becoming overprotective, which may limit the stroke survivor’s rehabilitation and community participation. The study concluded that a structured, client-centered falls prevention program targeting stroke survivors and caregivers is needed in Singapore to address these challenges and enhance recovery and reintegration into the community. |
| Lee at al. [41] | Enabling Stroke Rehabilitation in Home and Community Settings: A Wearable Sensor-Based Approach for Upper-Limb Motor Training | Digital support technologies | IEEE Journal of Translational Engineering in Health and Medicine | United States of America | To presents a novel technological approach that enables 1) detecting goal-directed upper limb movements during the performance of Activities of Daily Living (ADL), so that timely feedback can be provided to encourage the use of the affected limb, and 2) assessing the quality of motor performance during in-home rehabilitation exercises so that appropriate feedback can be generated to promote high-quality exercise. | Mixed methods | Stroke survivors,  health workers, community members | The results show that it is possible to detect 1) goal-directed movements during the performance of ADL with a c-statistic of 87.0% and 2) poorly performed movements in selected rehabilitation exercises with an F-score of 84.3%, thus enabling the generation of appropriate feedback. In a survey to gather preliminary data concerning the clinical adequacy of the proposed approach, 91.7% of occupational therapists demonstrated willingness to use it in their practice, and 88.2% of stroke survivors indicated that they would use it if recommended by their therapist. |
| Mainali et al. [42] | Feasibility and Efficacy of Nurse-Driven Acute Stroke Care | Digital support technologies | Journal of Stroke and Cerebrovascular Diseases | United States of America | To assess the feasibility of a nurse-driven acute stroke protocol using a parallel processing model. | Prospective, non-randomized feasibility study | Stroke survivors,  Health workers | The nurse-driven protocol for acute stroke care was feasible. Of the 57 Level 1 strokes (within 4.5 hours of symptom onset) that required acute stroke management, 78% were primarily nurse-driven. In telestroke encounters, 75% were also nurse-driven.  Nurse-driven protocols could effectively decrease critical time metrics in stroke care, making it beneficial in rural and community hospitals lacking stroke specialists. The average door-to-CT scan time was reduced from 38.9 minutes in non-nurse-driven cases to 24.4 minutes in nurse-driven cases (P < .04). The door-to-decision time was shorter in nurse-driven codes (43.5 minutes) compared to non-nurse-driven codes (149.6 minutes; P < .04). |
| Reeves et al. [43] | Michigan Stroke Transitions Trial A Clinical Trial to Improve Stroke Transitions | Digital support technologies | Circulation: Cardiovascular Quality and Outcomes | United States of America | To determine if providing home-based social worker-led case management (SWCM) or SWCM combined with a stroke-related information website could improve patient-reported outcomes for stroke survivors transitioning from hospital to home. | RCT | Stroke survivors | The combination of SWCM with access to the stroke-related website led to significant improvements in physical health and patient activation. However, there was no significant effect on mental health. Specifically, group-3 (SWCM + website) saw greater gains in physical health and patient activation compared to both the SWCM alone group and the usual care group. |
| Requena et al. [44] | Farmalarm Application for Mobile Devices Improves Risk Factor Control After Stroke | Digital support technologies | Stroke | Spain | Evaluate a smartphone-based platform aimed at increasing stroke patient awareness about the importance of healthy lifestyle, communication with healthcare providers, and adherence to treatment plans. | 2-arms, open-label non-randomized intervention study | Stroke survivors,  health workers | Mobile apps like Farmalarm are feasible for monitoring medication adherence and raising stroke awareness for stroke patients discharged home. It also appears to improve the management of vascular risk factors. After 90 days, patients in the Farmalarm group had a higher level of knowledge about vascular risk factors (86.0% versus 69.2%, P<0.01). A greater proportion of patients in the Farmalarm group had controlled diabetes (83.2% versus 63.5%, P<0.01) and hypercholesterolemia (80.3% versus 63.5%, P=0.03): 50.4% of patients in the Farmalarm group had all four risk factors under control, compared to 30.7% in the control group (P=0.02). A regression model indicated that the use of Farmalarm was independently associated with better control of all risk factors at 90 days (odds ratio 2.3; 95% CI, 1.14–4.6; P=0.02). |
| Cooray et al. [45] | Mobile Phone-Based Questionnaire for Assessing 3 Months Modified Rankin Score After Acute Stroke: A Pilot Study | Digital support technologies | Circulation: Cardiovascular Quality and Outcomes | Sweden | To investigate whether an automatic assessment of the modified Rankin Scale (mRS) using a mobile phone–based questionnaire could serve as an alternative to mRS assessments conducted at clinical visits after stroke. | Intervention study | Stroke survivors,  stroke caregivers | The mobile phone–based mRS assessment performed well compared to clinical visit assessments, with a 62.5% agreement between clinical visit and mobile mRS assessments, and a weighted kappa score of 0.89, indicating excellent concordance. The study concluded that mobile phone–based automatic assessments of mRS could serve as an alternative method for stroke follow-up, especially in resource-limited settings​. |
| Blanton et al. [46] | Content Validity and Satisfaction with a Caregiver Integrated Web-based Rehabilitation Intervention for Persons with Stroke | Digital support technologies | Topics in Stroke Rehabilitation | United States of America | To assess the content validity and satisfaction of a web-based intervention aimed at caregivers, intended to enhance stroke survivors' physical function and reduce negative outcomes for caregivers. | Mixed methods | Stroke caregivers,  health workers | On average, all caregivers agreed or strongly agreed that the modules were useful (4.42), easy to use (4.60) and acceptable (4.41). Mean total satisfaction score was 4.45, and average review time was 15 minutes per module. Expert reviewers agreed or strongly agreed that each module was accurate (4.95), feasible (4.8), easy to use (4.86) acceptable (4.96) and had appropriate problem relevance (4.65). |
| Caunca et al. [47] | Design and Usability Testing of the Stroke Caregiver Support System: A Mobile-Friendly Website to Reduce Stroke Caregiver Burden | Digital support technologies | Rehabilitation Nursing | United States of America | To design a mobile-friendly, internet-based website for stroke caregivers, to help reduce caregiver burden. | Iterative user-centered design study (Usability study) | Stroke caregivers | Caregivers appreciated the Stroke Caregiver Support System’s (SCSS) potential to reduce caregiver burden, particularly with its stress management resources and easy access via mobile devices. However, several areas for improvement were identified, such as the need for more engaging content, practical information, and greater customization of the modules. Further refinements were needed to maximize its usability and effectiveness. |
| Sureshkumar et al. [48] | Evaluation of the feasibility and acceptability of the ‘Care for Stroke’ intervention in India, a smartphone- enabled, carer-supported, educational intervention for management of disability following stroke | Digital support technologies | BMJ Open | India | To identify operational challenges of using the 'Care for Stroke' intervention.  To assess the feasibility and acceptability of the intervention. | Mixed methods | Stroke survivors,  stroke caregivers | Field-testing revealed operational issues with connectivity, video streaming, picture clarity, video quality, and app functionality. The intervention was revised and finalized before pilot testing. Pilot test results showed the 'Care for Stroke' intervention was feasible and acceptable in an Indian context, with over 90% (n=27) of participants finding it relevant, understandable, and useful. Additionally, over 96% (n=29) of stroke survivors and all caregivers (100%, n=30) rated it as excellent and highly useful. These results were further confirmed by qualitative interviews. |
| Zhou et al. [49] | Caregiver-Delivered Stroke Rehabilitation in Rural China The RECOVER Randomized Controlled Trial | Digital support technologies | Stroke | China | To evaluate the effectiveness of a novel, nurse-led, caregiver-delivered model of stroke rehabilitation, supported by a smartphone application. The primary goal was to improve the physical functioning of stroke patients, particularly in their basic self-care activities, as measured by the Barthel Index (BI), at 6 months post-intervention. | RCT | Stroke survivors,  Stroke caregivers,  Health workers | This study showed that there was no statistically significant difference in the adjusted 6-month Barthel Index scores between the intervention group and the control group (mean difference: -4.0, 95% CI: -10.0 to 2.9). Secondary outcome measures, including mobility and health-related quality of life, also showed no significant improvements. However, the process evaluation indicated that while the intervention was generally well-accepted by nurses, caregivers, and patients, it was perceived as too complex, leading to recommendations for future studies, such as the inclusion of community health workers and simpler, more frequent training sessions​. |
| Vloothuis et al. [50] | Description of the CARE4STROKE programme: A caregiver‐ mediated exercises intervention with e‐health support for stroke patients | Digital support technologies | Physiotherapy Research International | Netherland | The aim of the study is to describe the CARE4STROKE (C4S) program, which combines caregiver-mediated exercises (CME) with e-health support for stroke patients. The study intends to increase the intensity of practice and improve functional outcomes for stroke survivors while promoting early supported discharge (ESD), while reducing the burden on the caregiver. | Intervention study | Stroke survivors,  stroke caregivers,  health workers | The C4S program has the potential to offer a promising, cost-effective method for increasing the intensity of rehabilitation through caregiver involvement and e-health. Although the study is still in progress, preliminary findings show that the combination of CME and e-health can increase functional outcomes and improve mobility without increasing caregiver burden. The study reports that the intended 1,200 minutes of exercise time was almost fully achieved, suggesting successful adherence to the program. Additionally, caregiver strain and patient mobility improvements are closely monitored throughout the intervention​. |
| English et al. [51] | Reducing Sitting Time After Stroke: A Phase II Safety and Feasibility Randomized Controlled Trial | Digital support technologies | Archives of Physical Medicine and Rehabilitation | Australia | To evaluate the safety, feasibility, and effectiveness of reducing sitting time in stroke survivors, focusing on whether the intervention could decrease sitting time, particularly prolonged sitting, and increase standing, stepping, and moderate-to-vigorous physical activity​. | RCT | Stroke survivors; Stroke caregivers | The results indicated that the intervention was both safe and feasible, as no serious adverse events were reported, and adherence to the protocol was high. However, both the intervention group and the control group (which received counseling on calcium for bone health) reduced their sitting time and increased standing and stepping, with no significant differences between the two groups. The intervention group reduced sitting time by about 30 minutes per day, while the control group reduced it by about 40 minutes per day. This suggests that attention-matched control groups may contribute to positive outcomes, highlighting the importance of intervention design in clinical trials​. |
| Givon et al. [52] | Video-games used in a group setting is feasible and effective to improve indicators of physical activity in individuals with chronic stroke: a randomized controlled trial | Digital support technologies | Clinical Rehabilitation | Israel | To assess the practicality of using video games in a group setting and to compare their effectiveness against traditional group interventions for enhancing physical activity in individuals with chronic stroke. | RCT | Stroke survivors | Video games in small group settings are practical, safe, and enjoyable. They help improve physical activity measures in individuals with chronic stroke. Forty-seven participants (ages 29-78) with chronic stroke were randomly assigned to either a video game-based therapy group or a traditional therapy group. Both interventions showed high compliance rates (78% for video games, 66% for traditional therapy), but participant satisfaction was higher for the video game group (93%) compared to the traditional therapy group (71%) (χ(2)=4.98, P=0.026). No adverse effects were reported in either group. Both groups demonstrated significant improvements in gait speed (F=3.9, P=0.02), grip strength of the weaker hand (F=6.67, P=0.002), and stronger hand (F=7.5, P=0.001). However, there was no increase in daily steps or functional ability of the weaker hand in either group. |
| Paul et al. [53] | Increasing physical activity in stroke survivors using STARFISH, an interactive mobile phone application: a pilot study | Online stroke community system | Topics in Stroke Rehabilitation | United Kingdom | To evaluate the potential effectiveness of STARFISH, a mobile phone app-based intervention, which incorporates evidence-based behavior change techniques, in stroke survivors. | Non-randomized intervention study | Stroke survivors,  Community/society | STARFISH has the potential to improve physical activity and health outcomes in people after stroke. The average daily step count increased by 39.3% (4158 to 5791 steps/day) in the intervention group and reduced by 20.2% (3694 to 2947 steps/day) in the control group (p = 0.005 for group–time interaction). Similar patterns of data and group–time interaction were seen for walking time (p = 0.002) and fatigue (p = 0.003). There were no significant group–time interactions for other outcome measures. |
| Giachero et al. [54] | Conversational Therapy through Semi-Immersive Virtual Reality Environments for Language Recovery and Psychological Well- Being in Post Stroke Aphasia | Digital support technologies | Behavioural Neurology | Italy | To evaluate the effectiveness of conversational therapy delivered through semi-immersive virtual reality (VR) environments for enhancing language recovery and psychological well-being in individuals with chronic post-stroke aphasia. | RCT | Stroke survivors | There were no significant differences between the experimental group (VR therapy) and the control group (conventional therapy) in terms of overall outcomes, the VR group showed more widespread improvement across various cognitive and psychological areas. Specifically, within-group comparisons revealed significant improvements in language tasks (such as oral comprehension, repetition, and written language) and psychological measures (like self-esteem and mood) for the VR group, suggesting that VR therapy has broader benefits for aphasia rehabilitation compared to conventional therapy. |
| Cruickshank et al. [55] | Exploring the experiences of adults with stroke in virtual community-based stroke programs: a qualitative descriptive study | Online stroke community system | BMC Health Services Research | World-wide | To explore the experiences of stroke survivors in virtual community-based stroke programs, focusing on access, participation, and suggestions for improvement. | Qualitative study | Stroke survivors | Twelve participants (aged 32–69, 2–23 years post-stroke, eight women and four men) took part in this study. Five key themes emerged: (1) reasons for joining, such as peer connections and gaining knowledge; (2) barriers to participation, including technology challenges and inadequate facilitation; (3) facilitators to participation, such as remote access, platform features, and leader skills; (4) unmet needs, such as in-person interaction and personalized support; and (5) suggestions for improving program facilitation, content, and format. |
| Lo et al. [56] | Stroke survivor and caregiver experiences of virtual reality gaming to promote social participation: A qualitative study | Digital support technologies | Plos One | Hong Kong | To investigate stroke  survivors’ and caregivers’ perspectives of VR-based gaming rehabilitation modules for supporting  post-stroke recovery and social participation | Qualitative study | Stroke survivors; Stroke caregivers | Participants reported positive experiences with the VR-based gaming modules, including increased confidence in mobility and heightened awareness regarding outdoor safety and accessibility. Despite concerns regarding hygiene and discomfort with gaming equipment, participants found VR gaming to be engaging and conducive to their recovery. |
| Andrades-González et al. [57] | Perceptions and Attitudes of Informal Caregivers of Stroke Patients Regarding the Stroke-CareApp: A Phenomenological Study | Digital support technologies | Healthcare | Spain | To analyze the  opinions, perceptions, and attitudes of informal caregivers of stroke patients concerning the  use of m-health | Phenomenology | Stroke caregivers | M-health Impact on the caregiver, coping with caregiving, involvement  in caregiving, steps toward recovery in the absence of the caregiver, relevance for the caregiver, facilitating factors for the use of the app, source of consultation when in doubt and reliability of the information, and limitations in the use of the app |
| Demir and Gozum [58] | Evaluation of Quality, Content, and Use of the Web Site Prepared for Family Members Giving Care to Stroke Patients | Digital support technologies | Computers informaticsnursing: CIN | Turkey | Evaluate the quality,  content, usability, and efficacy of a Web site prepared for the purpose of improving the caregiving capability of family members who provide care for stroke survivors at home | Usability study | Stroke caregivers | The Web site quality and content were judged to be good and reliable to use. The Web site was efficiently used by caregivers. |
| De Simoni et al. [59] | Stroke survivors and their families receive information and support from an online forum: descriptive analysis of 2348 patients and qualitative study of a sample of participants. | Digital support technologies | BMJ Open | United Kingdom | To describe the characteristics of  participants of an online stroke forum, their reasons for  posting in the forum and whether responses addressed  users’ needs | Content analysis | Stroke survivors  Stroke caregivers | The main users’ intentions for writing in the forum were requests/offers of information and support (58%) and sharing their own experiences of stroke (35%). Most information needs were around stroke-related physical impairments, understanding the cause of stroke and the potential for recovery. Up to 95% of the users’ intentions were met by the replies received. |
| Eriksson et al. [60] | Family Members' Experiences of a Person-Centered Information and Communication Technology-Supported Intervention for Stroke Rehabilitation (F@ce 2.0): Qualitative Analysis | Digital support technologies | JMIR Rehabilitation and Assistive Technologies | Sweden | Describe family members’ experiences of the F@ce 2.0 (m-health) intervention from the perspective of  being a caregiver to a stroke survivor | Content analysis | Stroke caregivers | The main theme was the potential of F@ce 2.0 as a support for family members of stroke survivors in the sudden change of life. The categories were: dialogue and partnership with the F@ce 2.0 team, resuming daily activities lowers the demand for family support, support and involvement through the ICT component of F@ce 2.0, and engagement in F@ce 2.0, leading to suggestions for development. |
| Favilla et al. [61] | Personalized Video-Based Educational Platform to Improve Stroke Knowledge: A Randomized Clinical Trial | Digital support technologies | Journal of the American Heart Association | United States of America | To quantify the  impact of a personalized video- based educational platform to test the hypothesis that it improves patient satisfaction and  stroke knowledge | RCT | Stroke survivors; Stroke caregivers | The MyStroke personalized video- based education platform improved patient and caregiver satisfaction while improving some aspects of personalized stroke knowledge without improving general stroke knowledge |
| Firdaus et al. [62] | Explorative Survey on the Usage and Needs of Mobile Health Applications (mHealth) amongst Caregivers in Taking Care of Stroke Survivors | Digital support technologies | Medicine and Health | Malaysia | Explore the usage and needs of mHealth applications among  stroke caregivers and its associated factors | Cross-sectional observational study | Stroke caregivers | The overall usage of mHealth applications among stroke caregivers was high, with the need of specific types and features in mHealth applications among stroke caregivers |
| Firmawati et al. [63] | Mobile Health Application to Support Family Caregivers in Recurrent Stroke Prevention: Scoping Review | Digital support technologies | Open Access Macedonian Journal of Medical Sciences | World-wide | To identify existing mobile application designed to support family caregivers of  people with stroke disease | Review | Stroke caregivers | The results found three categories:caregivers’ support, involvement of caregivers in stroke care, and barriers. The majority of mobile applications were used to provide video education for caregivers including emotional care, nutrition, exercise, and recurrent stroke prevention. Poor connection was the biggest barrier to using mobile applications. |
| Gong et al. [64] | Development and Local Contextualization of Mobile Health Messages for Enhancing Disease Management Among Community-Dwelling Stroke Patients in Rural China: Multimethod Study | Digital support technologies | JMIR mHealth and uHhealth | China | To develop and pilot-test a mobile phone message–based  package, as a component of the SINEMA intervention | Mixed methods | Stroke survivors; Stroke caregivers | Voice messages containing simple and single-theme content, in plain language, with a repeated structure, a slow playback speed, and recorded in local dialect, were preferred by rural stroke patients. In addition, the dispatching algorithm and tools may also influence the acceptance of message-based interventions. |
| Kamwesiga et al. [65] | Experiences of using mobile phones in everyday life among persons with stroke and their families in Uganda | Digital support technologies | Disability and Rehabilitation | Uganda | To describe the experiences and meaning of using mobile phones in  everyday life after stroke, among persons with stroke and their family members | Grounded Theory | Stroke survivors; Stroke caregivers | Stroke survivors and caregivers described their mobile phone as enabling connection, belonging and agency across 7 themes: (1) enabling communication and connection; (2) a source of inspiration for agency; (3) to structure routine and activities; (4) as a facilitator of wellbeing; (5) to promote belonging and participation in relationships; (6) a facilitator of reintegration; and (7) as enabling family members to feel secure. The study gives support for the possibility of using mobile phones to facilitate change and community integration in the rehabilitation process after stroke. |
| Lobo et al. [66] | mHealth applications to support caregiver needs and engagement during stroke recovery: A content review | Digital support technologies | Research in Nursing and Health | World-wide | To investigate mHealth apps in supporting stroke caregiving  engagement based on three aspects: motivation, value, and satisfaction | Review | Stroke survivors; Stroke caregivers | Forty‐seven apps were included in this review that enabled caregivers to support their needs, such as adjustment to new roles and relationships, involvement in care and caring for oneself using several different functionalities. These functionalities include information resources, risk assessment, remote monitoring, data sharing, reminders and so on. However, no single app was identified that focuses on all aspects of stroke caregiving. We also identified several challenges faced by users through their reviews and the factors associated with  value and satisfaction |
| Kechik et al. [67] | A narrative review on mobile health (mHealth) app for stroke care and rehabilitation intervention for Malaysia | Digital support technologies | Malaysian Journal of Medical Science | Malaysia | To explore evidences about the digital health in stroke care and rehabilitation, especially for home-based care in Malaysia, including the current status and the availability of intervention modules | Review | Stroke survivors; Stroke caregivers | Study results indicate significant need for caregiver support after stroke, and mobile applications demonstrate significant promise in providing support. Key areas include: adherence to rehabilitation regimes; enhancing caregiver involvement; and reduction of logistical challenges. Ongoing challenges include digital literacy, equitable access, and integration within health care systems. The need for such interventions is especially pressing in Malaysia. |
| Pereira et al. [68] | Building Bridges between People with Stroke, Families, and Health Professionals: Development of a Blended Care Program for Self-Management | Digital support technologies | Journal of Clinical Medicine | Portugal | To develop a person-centered and tailored blended care program for post-stroke self-  management, taking into account the existing evidence-informed interventions and the perspectives  of Portuguese people with stroke, caregivers, and health professionals | Co-design | Stroke survivors; Stroke caregivers; Health workers | Stakeholder consultations revealed the need for (i) Personalized support and (ii) Building Bridges through small steps. The co-production stage developed the ComVida program by combining in-person and digital approaches, supported by a workbook and a mobile app. The evaluation of the resulting prototypes demonstrated a strong level of quality, understandability and actionability. The app also showed good usability (A-grade) and high levels of recommendation (5 stars). |
| Newland et al. [69] | Use of Video Education with Hospitalized Acute Stroke Patients: A Literature Review | Digital support technologies | MEDSURG Nursing | World-wide | To explore the role of video education to increase patients’ stroke knowledge and prevent further stroke events | Review | Stroke survivors; Health workers | Video technology and  printed educational resources, and taking time to talk with patients and their informal caregivers, are important strategies to enhance knowledge and understanding of home care management after stroke. Involving informal caregivers helps ensure adherence to recommended home care management interventions and enhances understanding of the need to follow post-stroke health recommendations provided by healthcare providers. |
| Nichols et al. [70] | Assessing Mobile Health Capacity and Task Shifting Strategies to Improve Hypertension Among Ghanaian Stroke Survivors | Digital support technologies | American Journal of the Medical Sciences | Ghana | To explore the barriers, facilitators and recommended mHealth  intervention strategies to control HTN in poststroke survivors | Concurrent triangulation design (Mixed methods) | Stroke survivors, Stroke caregivers, Clinicians,  Health workers,  Community | Findings support mHealth strategies for poststroke care delivery and HTN management and for task-shifting through a nurse-led model. Findings also identified barriers to care delivery and medication adherence across all levels of the social ecological model. |
| Siegel et al. [71] | A feasibility pilot using a mobile personal health assistant (PHA) app to assist stroke patient and caregiver communication after hospital discharge | Digital support technologies | mHealth | United States of America | To examine the potential of a PHA app to support ischemic stroke patients and improve post discharge satisfaction and decrease rehospitalization | Prospective observational study | Stroke survivors | Two of 21 eligible patients were enrolled in the study. Those who used the app were very satisfied with the personal health assistant (PHA) and their posthospital care coordination. Significant barriers to enrolment in the study give insights into the access challenges for stroke survivors in eHealth interventions. |
| Smith et al. [72] | Emotional adjustment post-stroke: A qualitative study of an online stroke community | Online stroke community system | Neuropsychological Rehabilitation | United Kingdom | To explore the process of emotional adjustment post-stroke and investigate the role played by participation in an online stroke community | Thematic analysis | Stroke survivors; Stroke caregivers | Findings described the trajectory of emotional recovery after stroke, and highlighted that trajectories varied between negative and positive pathways based on “trigger events”. These are not linked to chronological time. There is a need to improve awareness of emotional adjustment and their “triggers” amongst stroke survivors, carers and clinicians. |
| Tsang et al. [73] | Stroke caregivers’ perception on instant messaging application  use for psychological intervention: a qualitative study | Digital support technologies | Psychology, Health & Medicine | Hong Kong | To explore stroke caregivers’  perceived potential utility of IM-delivered psychological  intervention | Interpretive study | Stroke caregivers | Stroke caregivers have a strong need for personalised psychological support, and there is strong potential for IM-delivered psychological interventions emerged: this is linked to perceived high convenience and ease of use, perceived advantages that overcome existing barriers to services and message delivery tailored to individual needs. |
| Thompson et al. [74] | Mobile Technology–Based Interventions for Stroke Self-Management Support: Scoping Review | Digital support technologies | JMIR mHealth uHhealth | World-wide | To identify and describe the types of poststroke mHealth interventions evaluated using a randomized controlled trial design,  To determine whether (and how) such interventions align with well-accepted conceptualizations of self-management support  To identify the mHealth functions that facilitate self-management. | Review | Stroke survivors Stroke caregivers, Health workers | The 26 identified interventions addressed 7 focal areas (physical exercise, risk factor management, linguistic exercise, activities of daily living training, medication adherence, stroke education, and weight management), 5 types of mobile devices (mobile phones or smartphones, tablets, wearable sensors, wireless monitoring devices, and laptops), and 7 mHealth functions (educating, communicating, goal setting, monitoring, providing feedback, reminding, and motivating). Collectively, these interventions aligned well with the concept of self-management support. However, on an individual basis (per intervention), the alignment was less strong. Future interventions should be based on theory, include multidisciplinary approaches, and have breadth of scale. |
| Smythe et al. [75] | Access to health care for people with stroke in South Africa: a qualitative study of community perspectives | Community functions and roles | BMC Health Services Research | South Africa | To explore the perspectives and experiences of people with stroke (PWS) related to stroke care services to inform health system strengthening measures | Descriptive study | Stroke survivors, Stroke caregivers, health workers, community | Strategic leadership, governance and better resources at multiple levels are required to address the unmet demands and needs for health care of PWS. Stroke care could be strengthened by service providers routinely providing information about prevention and symptoms of stroke, treatment, and services to patients and their social support network. The role of family members in continuity of care could be strengthened by raising awareness of existing resources and referral pathways, and facilitating connections within services. |
| Sauvé-Schenk et al. [76] | A systematic review of social service and community resource interventions following stroke | Community functions and roles | Disability and Rehabilitation | World-wide | To identify and review interventions that aimed to increase access and use of finding and accessing social services and community resources post stroke | Review | Stroke survivors; Stroke caregivers, Community | The interventions included in this review varied in terms of target group, timing, and type of support provided (passive or active tailored information provision, referral service, navigation assistance). Outcome measures, for social service and community resource access, included discharge preparedness measures, service counts, observations, satisfaction evaluations, interviews, and open-ended questions. Stroke survivors and caregivers benefit from information, referral and ongoing support interventions |
| Sauerzopf et al. [77] | Technology Use for Home-Based Stroke Rehabilitation in Switzerland From the Perspectives of Persons Living With Stroke, Informal Caregivers, and Therapists: Qualitative Interview and Focus Group Study | Community functions and roles | JMIR Rehabilitation and Assistive Technologies | Switzerland | To explore the experiences and needs of persons living with stroke, informal caregivers, and therapists in using technology-based tools in a home environment for stroke rehabilitation in Switzerland | Descriptive study | Stroke survivors, Stroke caregivers, Health workers, Community | Four categories identified were: accessibility to quality rehabilitation, adaptability to patient differences, accountability or compliance with rehabilitation, and engagement with rehabilitation. Persons living with stroke used various tools within their rehabilitation process depending on their specific needs, and - while there are many tools available - sometimes felt overwhelmed with the selection process. Informal caregivers generally felt underserved and insufficiently informed throughout the rehabilitation process. They use technology-based tools to support their relatives affected by stroke in becoming more independent. Therapists appreciate the numerous possible applications of technology-based tools in rehabilitation, but this is constrained by a lack of clarity in Switzerland regarding cost coverage, recommendations, and training opportunities. |

**References**

1. Gooch HJ, Jarvis KA, et al. Behavior Change Approaches in Digital Technology–Based Physical Rehabilitation Interventions Following Stroke: Scoping Review. *Journal of Medical Internet Research*. 2024;26:e48725. doi:10.2196/48725
2. Reszel J, van den Hoek J, Nguyen T, et al. How Community-Based Teams Use the Stroke Recovery in Motion Implementation Planner: Longitudinal Qualitative Field Test Study. *JMIR Formative Research*. 2022;6(7):e37243. doi:10.2196/37243
3. Pindus DM, Mullis R, Lim L, et al. Stroke survivors' and informal caregivers' experiences of primary care and community healthcare services: A systematic review and meta-ethnography. *PLoS One*. 2018;13(2):e0192533. doi:10.1371/journal.pone.0192533
4. White J, Magin P, Attia J, Sturm J, McElduff P, Carter G. Predictors of health-related quality of life in community-dwelling stroke survivors: a cohort study. *Family Practice*. 2016;33(4):382–387. doi:10.1093/fampra/cmw011
5. Feigin VL, Owolabi MO, World Stroke Organization–Lancet Neurology Commission Stroke Collaboration Group. Pragmatic solutions to reduce the global burden of stroke: a World Stroke Organization–Lancet Neurology Commission. *Lancet Neurology*. 2023;22(12):1160–1206. doi:10.1016/S1474-4422(23)00277-6
6. Fu B, Mei Y, Lin B, et al. Effects of a Benefit-Finding Intervention in Stroke Caregivers in Communities. *Clinical Gerontologist*. 2022;45(5):1317–1329. doi:10.1080/07317115.2020.1765062
7. Cameron JI, Gignac MAM. “Timing It Right”: A conceptual framework for addressing the support needs of family caregivers to stroke survivors from the hospital to the home. *Patient Education and Counseling*. 2008;70(3):305–314. doi:10.1016/j.pec.2007.10.020
8. Lobo EH, Abdelrazek M, Grundy J, et al. Caregiver Engagement in Stroke Care: Opportunities and Challenges in Australia and Denmark. *Frontiers in Public Health*. 2021;9:758808. doi:10.3389/fpubh.2021.758808
9. Leonardi M, Fheodoroff K. Goal Setting with ICF (International Classification of Functioning, Disability and Health) and Multidisciplinary Team Approach in Stroke Rehabilitation. In: Platz T, ed. *Clinical Pathways in Stroke Rehabilitation: Evidence-Based Clinical Practice Recommendations*. Cham: Springer; 2021:35–56. doi:10.1007/978-3-030-58505-1_3
10. Aravind G, Bashir K, Cameron JI, et al. Community-based exercise programs incorporating healthcare-community partnerships to improve function post-stroke: feasibility of a 2-group randomized controlled trial. *Pilot and Feasibility Studies*. 2022;8(1):88. doi:10.1186/s40814-022-01037-9
11. Smith CE, Levonian Z, Ma H, Giaquinto R, Lein-McDonough G, Li Z, O’Connor-Von S, Yarosh S. “I Cannot Do All of This Alone”: Exploring Instrumental and Prayer Support in Online Health Communities. *ACM Transactions on Computer-Human Interaction*. 2020;27(5):1–41. doi:10.1145/3402855
12. Magwood GS, Nichols M, Jenkins C, Logan A, Qanungo S, Zigbuo-Wenzler E, Ellis C. Community-Based Interventions for Stroke Provided by Nurses and Community Health Workers: A Review of the Literature. *Journal of Neuroscience Nursing*. 2020;52(4):152–159. doi:10.1097/JNN.0000000000000512
13. Wright WJA, Howdle C, Coulson NS, De Simoni A. Exploring the Types of Social Support Exchanged by Survivors of Pediatric Stroke and Their Families in an Online Peer Support Community: Qualitative Thematic Analysis. *Journal of Medical Internet Research*. 2024;26:e49440. doi:10.2196/49440
14. Millar DJ, Mason H, Kidd L, IMPETUS Research Team. What is important in supporting self-management in community stroke rehabilitation? A Q methodology study. *Disability and Rehabilitation*. 2023;45(14):2307–2315. doi:10.1080/09638288.2022.2087766
15. Deutschbein J, Grittner U, Schneider A, Schenk L. Community care coordination for stroke survivors: results of a complex intervention study. *BMC Health Services Research*. 2020;20(1):954. doi:10.1186/s12913-020-05993-x
16. Norlander A, Iwarsson S, Jönsson AC, Lindgren A, Lexell EM. Participation in social and leisure activities while re-constructing the self: understanding strategies used by stroke survivors from a long-term perspective. *Disability and Rehabilitation*. 2021;44(16):4284–4292. doi:10.1080/09638288.2021.1900418
17. Thomas K, Gamlin C, De Simoni A, Mullis R, Mant J. How is poststroke fatigue understood by stroke survivors and carers? A thematic analysis of an online discussion forum. *BMJ Open*. 2019;9:e028958. doi:10.1136/bmjopen-2018-028958
18. Scrivener K, Sewastenko J, Bouvier-Farrell A, MacDonald K, Van Rijn T, Tezak J, et al. Feasibility of a self-managed, video-guided exercise program for community-dwelling people with stroke. *Stroke Research and Treatment*. 2021;2021:5598100. doi:10.1155/2021/5598100
19. Olafsdottir S, et al. Developing ActivABLES for community-dwelling stroke survivors using the Medical Research Council framework for complex interventions. *BMC Health Services Research*. 2020;20:784. doi:10.1186/s12913-020-05673-w
20. Lee M, Son J, Kim J, Pyun SB, Eun SD, Yoon BC. Comparison of individualized virtual reality– and group-based rehabilitation in older adults with chronic stroke in community settings: a pilot randomized controlled trial. *European Journal of Integrative Medicine*. 2016;8(5):738–746. doi:10.1016/j.eujim.2016.08.012
21. Olafsdottir S, et al. Feasibility of ActivABLES to promote home-based exercise and physical activity of community-dwelling stroke survivors with support from caregivers: a mixed methods study. *BMC Health Services Research*. 2021;21:562. doi:10.1186/s12913-021-06507-5
22. Camicia M, Lutz B, Summers D, Klassman L, Vaughn S. Nursing's role in successful stroke care transitions across the continuum: from acute care into the community. *Stroke*. 2021;52(12):e794–e805. doi:10.1161/STROKEAHA.121.033938
23. Demers M, Bishop L, Cain A, et al. Wearable technology to capture arm use of people with stroke in home and community settings: feasibility and early insights on motor performance. *Physical Therapy*. 2024;104(2):pzad172. doi:10.1093/ptj/pzad172
24. Freund M, Carey M, Dilworth S, et al. Effectiveness of information and communications technology interventions for stroke survivors and their support people: a systematic review. *Disability and Rehabilitation*. 2022;44(17):4563–4578. doi:10.1080/09638288.2021.1913245
25. Johnson L, Bird ML, Muthalib M, Teo WP. An innovative STRoke Interactive Virtual thErapy (STRIVE) online platform for community-dwelling stroke survivors: a randomized controlled trial. *Archives of Physical Medicine and Rehabilitation*. 2020;101(7):1131–1137. doi:10.1016/j.apmr.2020.03.011
26. Berkeley SBJ, Johnson AM, Mormer ER, et al. Referral to community-based rehabilitation following acute stroke: findings from the COMPASS pragmatic trial. *Circulation: Cardiovascular Quality and Outcomes*. 2024;17(1):e010026. doi:10.1161/CIRCOUTCOMES.123.010026
27. Juengst SB, et al. Use of mHealth technology for patient-reported outcomes in community-dwelling adults with acquired brain injuries: a scoping review. *International Journal of Environmental Research and Public Health*. 2022;19(3):1623. doi:10.3390/ijerph19031623
28. Kerr A, Smith M, Reid L, Baillie L. Adoption of stroke rehabilitation technologies by the user community: qualitative study. *JMIR Rehabilitation and Assistive Technologies*. 2018;5(2):e15. doi:10.2196/rehab.9219
29. Krishnan S, Mandala MA, Wolf SL, Howard A, Kesar TM. Perceptions of stroke survivors regarding factors affecting adoption of technology and exergames for rehabilitation. *PM&R*. 2023;15(11):1403–1410. doi:10.1002/pmrj.12963
30. Lam LCW, et al. Evaluation of cost-effectiveness of a virtual multidisciplinary stroke care clinic for community-dwelling survivors of stroke. *Journal of the American Heart Association*. 2023;12(6):e028123. doi:10.1161/JAHA.122.028123
31. Lobo EH, Frølich A, Rasmussen LJ, et al. Understanding the methodological issues and solutions in the research design of stroke caregiving technology. *Frontiers in Public Health*. 2021;9:647249. doi:10.3389/fpubh.2021.647249
32. Lo SHS, Chau JPC, Lau AYL, et al. Virtual multidisciplinary stroke care clinic for community-dwelling stroke survivors: a randomized controlled trial. *Stroke*. 2023;54(10):2482–2490. doi:10.1161/STROKEAHA.123.043605
33. Luo Z, Lim AE, Durairaj P, Tan KK, Verawaty V. Development of a compensation-aware virtual rehabilitation system for upper extremity rehabilitation in community-dwelling older adults with stroke. *Journal of NeuroEngineering and Rehabilitation*. 2023;20(1):56. doi:10.1186/s12984-023-01183-y
34. Murakami K, Tsubota-Utsugi M, Satoh M, et al. Impaired higher-level functional capacity as a predictor of stroke in community-dwelling older adults: the Ohasama Study. *Stroke*. 2016;47(2):323–328. doi:10.1161/STROKEAHA.115.011131
35. Peters DM, O’Brien ES, Kamrud KE, et al. Utilization of wearable technology to assess gait and mobility post-stroke: a systematic review. *Journal of NeuroEngineering and Rehabilitation*. 2021;18(1):67. doi:10.1186/s12984-021-00863-x
36. Sarfo FS, Obiako R, Nichols M, et al. Knowledge and perspectives of community members on risk assessment for stroke prevention using mobile health approaches in Nigeria. *Journal of Stroke and Cerebrovascular Diseases*. 2023;32(9):107265. doi:10.1016/j.jstrokecerebrovasdis.2023.107265
37. Saywell NL, Vandal AC, Mudge S, et al. Telerehabilitation after stroke using readily available technology: a randomized controlled trial. *Neurorehabilitation and Neural Repair*. 2021;35(1):88–97. doi:10.1177/1545968320971765
38. Silvera-Tawil D, Cameron J, Li J, et al. Multicomponent support program for secondary prevention of stroke using digital health technology: co-design study with people living with stroke or transient ischemic attack. *Journal of Medical Internet Research*. 2024;26:e54604. doi:10.2196/54604
39. Sun X, Ding J, Dong Y, Ma X, et al. A survey of technologies facilitating home and community-based stroke rehabilitation. *International Journal of Human–Computer Interaction*. 2022;39(5):1016–1042. doi:10.1080/10447318.2022.2050545
40. Xu T, O’Loughlin K, Clemson L, Lannin NA, Dean C, Koh G. Developing a falls prevention program for community-dwelling stroke survivors in Singapore: client and caregiver perspectives. *Disability and Rehabilitation*. 2019;41(9):1044–1054. doi:10.1080/09638288.2017.1419293
41. Lee M, Son J, Kim J, Pyun SB, Eun SD, Yoon BC. Enabling stroke rehabilitation in home and community settings: a wearable sensor-based approach for upper-limb motor training. *IEEE Journal of Translational Engineering in Health and Medicine*. 2018;6:1–11. doi:10.1109/JTEHM.2018.2799611
42. Mainali S, Stutzman S, Sengupta S, Dirickson A, Riise L, Jones D, Yang J, Olson DM. Feasibility and efficacy of nurse-driven acute stroke care. *Journal of Stroke and Cerebrovascular Diseases*. 2017;26(5):987–991. doi:10.1016/j.jstrokecerebrovasdis.2016.11.007
43. Reeves MJ, Fritz MC, Woodward AT, et al. Michigan Stroke Transitions Trial: a clinical trial to improve stroke transitions. *Circulation: Cardiovascular Quality and Outcomes*. 2019;12:e005493. doi:10.1161/CIRCOUTCOMES.118.005493
44. Requena M, Montiel E, Baladas M, et al. Farmalarm: application for mobile devices improves risk factor control after stroke. *Stroke*. 2019;50(7):1819–1824. doi:10.1161/STROKEAHA.118.023795
45. Cooray C, Matusevicius M, Wahlgren N, Ahmed N. Mobile phone-based questionnaire for assessing 3-month modified Rankin score after acute stroke: a pilot study. *Circulation: Cardiovascular Quality and Outcomes*. 2015;8(2):125–130. doi:10.1161/CIRCOUTCOMES.114.001444
46. Blanton S, Dunbar S, Clark PC. Content validity and satisfaction with a caregiver-integrated web-based rehabilitation intervention for persons with stroke. *Topics in Stroke Rehabilitation*. 2018;25(3):168–173. doi:10.1080/10749357.2017.1419618
47. Caunca MR, Simonetto M, Hartley G, Wright CB, Czaja SJ. Design and usability testing of the stroke caregiver support system: a mobile-friendly website to reduce stroke caregiver burden. *Rehabilitation Nursing*. 2020;45(3):166–177. doi:10.1097/RNJ.0000000000000196
48. Sureshkumar K, Murthy G, Natarajan S, Naveen C, Goenka S, Kuper H. Evaluation of the feasibility and acceptability of the “Care for Stroke” intervention in India. *BMJ Open*. 2016;6:e009243. doi:10.1136/bmjopen-2015-009243
49. Zhou B, Zhang J, Zhao Y, Li X, Anderson CS, Xie B, et al. Caregiver-delivered stroke rehabilitation in rural China: the RECOVER randomized controlled trial. *Stroke*. 2019;50(7):1825–1830. doi:10.1161/STROKEAHA.118.021558
50. Vloothuis J, de Bruin J, Mulder M, Nijland R, Kwakkel G, van Wegen EEH. Description of the CARE4STROKE programme: a caregiver-mediated exercises intervention with e-health support for stroke patients. *Physiotherapy Research International*. 2018;23(2):e1719. doi:10.1002/pri.1719
51. English C, Healy GN, Olds T, Parfitt G, Borkoles E, Coates A, et al. Reducing sitting time after stroke: a phase II safety and feasibility randomized controlled trial. *Archives of Physical Medicine and Rehabilitation*. 2016;97(2):273–280. doi:10.1016/j.apmr.2015.10.099
52. Givon N, Zeilig G, Weingarden H. Video-games used in a group setting are feasible and effective to improve indicators of physical activity in individuals with chronic stroke: a randomized controlled trial. *Clinical Rehabilitation*. 2016;30(4):383–392. doi:10.1177/0269215515582474
53. Paul L, Wyke S, Brewster S, Sattar N, Gill JM, Alexander G, et al. Increasing physical activity in stroke survivors using STARFISH, an interactive mobile phone application: a pilot study. *Topics in Stroke Rehabilitation*. 2016;23(3):170–177. doi:10.1080/10749357.2015.1122266
54. Giachero A, Calati M, Pia L, La Vista L, et al. Conversational therapy through semi-immersive virtual reality environments for language recovery and psychological well-being in post-stroke aphasia. *Behavioural Neurology*. 2020;2020:2846046. doi:10.1155/2020/2846046
55. Cruickshank A, Brooks ED, Sperling C, et al. Exploring the experiences of adults with stroke in virtual community-based stroke programs: a qualitative descriptive study. *BMC Health Services Research*. 2024;24:600. doi:10.1186/s12913-024-11043-7
56. Lo SHS, Chau JPC, Lau AYL, et al. Stroke survivor and caregiver experiences of virtual reality gaming to promote social participation: a qualitative study. *PLoS One*. 2023;18(2):e0281234. doi:10.1371/journal.pone.0281234
57. Andrades-González I, Rodríguez-Estrabot N, Magdaleno-Moya R, Molina-Mula J. Perceptions and attitudes of informal caregivers regarding the Stroke-CareApp: a phenomenological study. *Healthcare*. 2025;13:2082. doi:10.3390/healthcare13172082
58. Demir Y, Gozum S. Evaluation of quality, content, and use of the website prepared for family caregivers of stroke patients. *Computers, Informatics, Nursing*. 2015;33(9):396–403. doi:10.1097/CIN.0000000000000165
59. De Simoni A, Shanks A, Balasooriya-Smeekens C, Mant J. Stroke survivors and their families receive information and support from an online forum: descriptive analysis and qualitative study. *BMJ Open*. 2016;6(4):e010501. doi:10.1136/bmjopen-2015-010501
60. Eriksson G, Söderhielm K, Erneby M, Guidetti S. Family members' experiences of a person-centered ICT-supported intervention for stroke rehabilitation (F@ce 2.0): qualitative analysis. *JMIR Rehabilitation and Assistive Technologies*. 2025;12:e69878. doi:10.2196/69878
61. Favilla CG, Reehal N, Cummings SR, Burdett R, Stein LA, Shakibajahromi B, Yuan K, Sloane KL, Kasner SE. Personalized Video-Based Educational Platform to Improve Stroke Knowledge: A Randomized Clinical Trial. *Journal of the American Heart Association*. 2024;13(15):e035176. doi:10.1161/JAHA.124.035176
62. Firdaus A, Noor AAA, Wan AWZ, et al. Explorative Survey on the Usage and Needs of Mobile Health Applications (mHealth) amongst Caregivers in Taking Care of Stroke Survivors. *Medicine & Health*. 2022;17(2):85–100. doi:10.17576/MH.2022.1702.07
63. Firmawati E, Setyopanoto I, Pangastuti HS. Mobile Health Application to Support Family Caregivers in Recurrent Stroke Prevention: Scoping Review. *Open Access Macedonian Journal of Medical Sciences*. 2022;9(5):142–151. doi:10.3889/oamjms.2022.7859
64. Gong E, Gu W, Luo E, Tan L, Donovan J, Sun C, Yang Y, Zang L, Bao P, Yan LL. Development and Local Contextualization of Mobile Health Messages for Enhancing Disease Management Among Community-Dwelling Stroke Patients in Rural China: Multimethod Study. *JMIR mHealth and uHealth*. 2019;7(12):e15758. doi:10.2196/15758
65. Kamwesiga JT, Tham K, Guidetti S. Experiences of using mobile phones in everyday life among persons with stroke and their families in Uganda: a qualitative study. *Disability and Rehabilitation*. 2017;39(5):438–449. doi:10.3109/09638288.2016.1146354
66. Lobo EH, Frølich A, Kensing F, Rasmussen LJ, Livingston PM, Grundy J, Abdelrazek M. mHealth applications to support caregiver needs and engagement during stroke recovery: a content review. *Research in Nursing & Health*. 2021;44(1):213–225. doi:10.1002/nur.22096
67. Kechik LTTSM, Musa KI, Abdullah JM, Kamalakannan S, Sidek NN, Hamzah N, et al. A narrative review on mobile health (mHealth) app for stroke care and rehabilitation intervention for Malaysia. *Malaysian Journal of Medical Sciences*. 2025;32(3):49–72. doi:10.21315/mjms-03-2025-154
68. Pereira CM, Matos M, Carvalho D, Macedo P, Calheiros JM, Alves J, Ferreira PL, et al. Building Bridges between People with Stroke, Families, and Health Professionals: Development of a Blended Care Program for Self-Management. *Journal of Clinical Medicine*. 2024;13:300. doi:10.3390/jcm13010300
69. Newland P, Sargent R, Van Aman MN, Hamilton K, Hendricks-Ferguson V. Use of Video Education with Hospitalized Acute Stroke Patients: A Literature Review. *MEDSURG Nursing*. 2023;32:106.
70. Nichols M, Sarfo FS, Singh A, et al. Assessing Mobile Health Capacity and Task Shifting Strategies to Improve Hypertension Among Ghanaian Stroke Survivors. *American Journal of the Medical Sciences*. 2017;354(6):573–580. doi:10.1016/j.amjms.2017.08.005
71. Siegel J, Edwards E, Mooney L, Smith C, Peel JB, Dole A, Maler P, Freeman WD. A feasibility pilot using a mobile personal health assistant (PHA) app to assist stroke patient and caregiver communication after hospital discharge. *mHealth*. 2016;2:31. doi:10.21037/mhealth.2016.08.02
72. Smith FE, Jones C, Gracey F, Mullis R, Coulson NS, De Simoni A. Emotional adjustment post-stroke: a qualitative study of an online stroke community. *Neuropsychological Rehabilitation*. 2021;31(3):414–431. doi:10.1080/09602011.2019.1702561
73. Tsang WN, Lee JJ, Yang SC, Poon JCY, Lau EYY. Stroke caregivers' perception on instant messaging application use for psychological intervention: a qualitative study. *Psychology, Health & Medicine*. 2024;29(7):1208–1221. doi:10.1080/13548506.2024.2332925
74. Thompson AN, Dawson DR, Legasto-Mulvale JM, Chandran N, Tanchip C, Niemczyk V, Rashkovan J, Jeyakumar S, Wang RH, Cameron JI, Nalder E. Mobile Technology–Based Interventions for Stroke Self-Management Support: Scoping Review. JMIR Mhealth Uhealth 2023;11:e46558. doi: [10.2196/46558](https://doi.org/10.2196/46558)
75. Smythe T, Inglis-Jassiem G, Conradie T, et al. Access to health care for people with stroke in South Africa: a qualitative study of community perspectives. *BMC Health Services Research*. 2022;22:464. doi:10.1186/s12913-022-07903-9
76. Sauvé-Schenk K, Duong P, Savard J, Durand F. A systematic review of social service and community resource interventions following stroke. *Disability and Rehabilitation*. 2022;44(13):2948–2957. doi:10.1080/09638288.2020.1851780
77. Sauerzopf L, Luft A, Maeusli V, Klamroth-Marganska V, Sy M, Spiess MR. Technology Use for Home-Based Stroke Rehabilitation in Switzerland From the Perspectives of Persons Living With Stroke, Informal Caregivers, and Therapists: Qualitative Interview and Focus Group Study. *JMIR Rehabilitation and Assistive Technologies*. 2024;11:e59781. doi:10.2196/59781
