# Supplementary material for: Online Community Support for Stroke Survivors and Caregivers: Scoping Review
Source: J Med Internet Res. 2026 Apr 29;28:e71190. doi: 10.2196/71190 (PMC13127857; doi:10.2196/71190)
Supplement: Multimedia Appendix 3 [file jmir-v28-e71190-s003.docx]

**Journals Cited**

| **Journal** | **Total** |
| --- | --- |
| BMJ Open | 3 |
| Disability and Rehabilitation | 6 |
| Stroke | 5 |
| BMC Health Services Research | 5 |
| Circulation: Cardiovascular Quality and Outcomes | 3 |
| Journal of Medical Internet Research | 3 |
| Topics in Stroke Rehabilitation | 2 |
| Archives of Physical Medicine and Rehabilitation | 2 |
| Frontiers in Public Health | 2 |
| Journal of NeuroEngineering and Rehabilitation | 2 |
| Journal of Stroke and Cerebrovascular Diseases | 2 |
| ACM Transaction Computer Human Interaction | 1 |
| American Journal of the Medical Sciences | 1 |
| Behavioural Neurology | 1 |
| Clinical Gerontologist | 1 |
| Clinical Pathways in Stroke Rehabilitation | 1 |
| Clinical Rehabilitation | 1 |
| Computers, informatics, nursing : CIN | 1 |
| European Journal of Integrative Medicine | 1 |
| Health Service Research | 1 |
| Healthcare | 1 |
| International Journal of Environmental Research and Public Health | 1 |
| International Journal of Human–Computer Interaction | 1 |
| IEEE Journal of Translational Engineering in Health and Medicine | 1 |
| JMIR Formative Research | 1 |
| JMIR Rehabilitation and Assistive Technologies | 3 |
| JMIR Mhealth and Uhealth | 2 |
| Journal of the American Heart Association | 2 |
| Journal of Clinical Medicine | 1 |
| Journal of Neuroscience Nursing | 1 |
| Lancet Neurology Commissions | 1 |
| Malaysia Journal of Medical Science | 1 |
| Medicine and Health | 1 |
| MEDSURG Nursing | 1 |
| mHealth | 1 |
| Neurorehabilitation and Neural Repair | 1 |
| Neuropsychological Rehabilitation | 1 |
| Open Access Macedonian Journal of Medical Sciences | 1 |
| Patient Education and Counseling | 1 |
| Pilot and Feasibility Studies | 1 |
| Physical Therapy & Rehabilitation Journal | 1 |
| Psychology, health & medicine | 1 |
| Physiotherapy Research International | 1 |
| PLOSONE | 2 |
| PM&R (Physical Medicine and Rehabilitation) | 1 |
| Rehabilitation Nursing | 1 |
| Research in nursing & health | 1 |
| Stroke Research and Treatment | 1 |
