# Supplementary material for: Online Community Support for Stroke Survivors and Caregivers: Scoping Review
Source: J Med Internet Res. 2026 Apr 29;28:e71190. doi: 10.2196/71190 (PMC13127857; doi:10.2196/71190)
Supplement: Multimedia Appendix 4 [file jmir-v28-e71190-s004.docx]

## **Technologies Used to Support Stroke Survivors in the Online Community**

| **Technology** | **Function** | **Types of Support** | **References** |  |
| --- | --- | --- | --- | --- |
| Mobile health (m-health) | Clinicians can send digital education, community resources, tasks to complete, and surveys directly to the caregiver’s m-health. It can also be used to provide training for health survivors and caregivers using text messages, educational videos, and a workbook. Policy makers used m-health to support the stroke survivors’ rehabilitation process. | Informational support | [1, 2, 3, 4, 5, 6, 7, 8, 9, 10, 11, 12, 13, 14, 15] |  |
|  |  | Emotional support | [10, 15, 16, 17] |  |
|  |  | Social support | [1, 2, 7, 8, 15, 16, 18, 19, 20, 21] |  |
|  |  | Esteem support | [15] |  |
|  |  | Advice/knowledge | [1, 2, 3, 15, 16, 22] |  |
|  |  | Tangible aid | [1, 2, 3, 4, 6, 15, 16, 17, 18, 19, 23, 24, 25, 26] |  |
|  |  | Not mention the type of community support | [27] |  |
| EHR or EMR | Used by funders to analyze the sociodemographic data, health services and costs they cover for their clients. | Tangible aid | [28] |  |
| E-mail | Used in virtual community-based organization stroke programs to optimize participant experiences and outcomes. | Informational support | [29] |  |
|  |  | Advice/knowledge | [29] |  |
| Web-based system | Used for stroke surveillance services, allowing data in real time, improving data quality, and allowing better exchange of information. and to plan community-based exercise programs, such as conducting community assessment and developing referral pathways. | Informational support | [1, 4, 30, 31, 32, 33, 34, 35] |  |
|  |  | Social support | [30, 36, 37] |  |
|  |  | Emotional support | [31] |  |
|  |  | Advice/knowledge | [30, 37] |  |
|  |  | Tangible aid | [3, 4, 38] |  |
|  |  | Involvement in decision making | [2, 31] |  |
| Online forum | A two-way communications platform used by caregivers to provide support. | Informational support | [39, 40, 41] |  |
|  |  | Emotional support | [39, 41, 42] |  |
|  |  | Social support | [39, 40, 41] |  |
|  |  | Spiritual support | [39] |  |
|  |  | Advice/knowledge | [39] |  |
|  |  | Tangible aid | [39] |  |
| Telehealth or telerehabilitation or teleconsultation or telestroke | Clinicians can use this technology to send digital education, community resources, tasks to complete, and surveys. | Informational support | [43] |  |
|  |  | Social support | [43, 44] |  |
|  |  | Advice/knowledge | [43] |  |
|  |  | Tangible aid | [43, 45, 46] |  |
| Video-guided exercise app | Video-guided exercise app used to provide ongoing motivation and tips to keep the rehabilitation interesting. Health workers could provide advice, education about physical activity, and activities to enhance the skills required for self-management of physical activity. | Esteem support | [38] |  |
|  |  | Advice or knowledge | [2, 29, 47] |  |
|  |  | Tangible aid | [38, 48] |  |
| Communication technology such as video conferencing or IM app | Used in virtual community-based organization stroke programs to optimize participant experiences and outcomes. | Informational support | [29, 49] |  |
|  |  | Social support | [29] |  |
|  |  | Advice/knowledge | [29] |  |
| VR/AR | Used for physical stroke rehabilitation. | Informational support | [50, 51] |  |
|  |  | Social support | [15, 50, 51, 52] |  |
|  |  | Advice/knowledge | [15, 50, 52] |  |
|  |  | Tangible aid | [15, 48, 50, 52, 53, 54, 55, 56] |  |
| Sensors or wearable technology | Used for physical stroke rehabilitation. | Social support | [15, 16, 55, 57, 58] |  |
|  |  | Advice/knowledge | [15, 16] |  |
|  |  | Tangible aid | [6, 15, 16, 48, 55, 57, 59] |  |
|  |  | Not mention the type of community support | [60] |  |

**References**

1. Feigin VL, Owolabi MO; World Stroke Organization-Lancet Neurology Commission Stroke Collaboration Group. Pragmatic solutions to reduce the global burden of stroke: a World Stroke Organization-Lancet Neurology Commission. Lancet Neurol. 2023;22(12):1160–1206. doi:10.1016/S1474-4422(23)00277-6.
2. Camicia M, Lutz B, Summers D, Klassman L, Vaughn S. Nursing's Role in Successful Stroke Care Transitions Across the Continuum: From Acute Care Into the Community. Stroke. 2021;52(12):e794–e805. doi:10.1161/STROKEAHA.121.033938.
3. Lobo EH, Frølich A, Rasmussen LJ, et al. Understanding the Methodological Issues and Solutions in the Research Design of Stroke Caregiving Technology. Front Public Health. 2021;9:647249. doi:10.3389/fpubh.2021.647249.
4. Reeves MJ, Fritz MC, Woodward AT, et al. Michigan Stroke Transitions Trial. A clinical trial to improve stroke transitions. Circ Cardiovasc Qual Outcomes. 2019;12:e005493.
5. Cooray C, Matusevicius M, Wahlgren N, Ahmed N. Mobile Phone-Based Questionnaire for Assessing 3 Months Modified Rankin Score After Acute Stroke: A Pilot Study. Circ Cardiovasc Qual Outcomes. 2015;8:125–130.
6. English C, Healy GN, Olds T, et al. Reducing sitting time after stroke: a phase II safety and feasibility randomized controlled trial. Arch Phys Med Rehabil. 2016;97(2):273–280.
7. Andrades-González I, Rodríguez-Estrabot N, Magdaleno-Moya R, Molina-Mula J. Perceptions and Attitudes of Informal Caregivers of Stroke Patients Regarding the Stroke-CareApp: A Phenomenological Study. Healthcare. 2025;13:2082.
8. Eriksson G, Söderhielm K, Erneby M, Guidetti S. Family Members' Experiences of a Person-Centered ICT-Supported Intervention for Stroke Rehabilitation (F@ce 2.0): Qualitative Analysis. JMIR Rehabil Assist Technol. 2025;12:e69878.
9. Firdaus A, Noor AAA, Wan AWZ, et al. Explorative Survey on the Usage and Needs of Mobile Health Applications (mHealth) amongst Caregivers in Taking Care of Stroke Survivors. Med Health. 2022;17(2):85–100.
10. Firmawati E, Setyopanoto I, Pangastuti HS. Mobile Health Application to Support Family Caregivers in Recurrent Stroke Prevention: Scoping Review. Open Access Maced J Med Sci. 2022;9(5):142–151.
11. Lobo EH, Frølich A, Kensing F, Rasmussen LJ, et al. mHealth applications to support caregiver needs and engagement during stroke recovery: A content review. Res Nurs Health. 2021;44(1):213–225.
12. Kechik LTTSM, Musa KI, Abdullah JM, et al. A narrative review on mobile health (mHealth) app for stroke care and rehabilitation intervention for Malaysia. Malays J Med Sci. 2025;32(3):49–72.
13. Nichols M, Sarfo FS, Singh A, et al. Assessing Mobile Health Capacity and Task Shifting Strategies to Improve Hypertension Among Ghanaian Stroke Survivors. Am J Med Sci. 2017;354(6):573–580.
14. Siegel J, Edwards E, Mooney L, et al. A feasibility pilot using a mobile personal health assistant (PHA) app to assist stroke patient and caregiver communication after hospital discharge. mHealth. 2016;2:31.
15. Olafsdottir SA, Jonsdottir H, Bjartmarz I, et al. Feasibility of ActivABLES to promote home-based exercise and physical activity of community-dwelling stroke survivors: a mixed methods study. BMC Health Serv Res. 2020;20:562.
16. Olafsdottir SA, Jonsdottir H, Magnusson C, et al. Developing ActivABLES for community-dwelling stroke survivors using the Medical Research Council framework. BMC Health Serv Res. 2020;20:463.
17. Juengst SB, Terhorst L, Nabasny A, et al. Use of mHealth Technology for Patient-Reported Outcomes in Community-Dwelling Adults with Acquired Brain Injuries: A Scoping Review. Int J Environ Res Public Health. 2021;18(4):2173.
18. Sarfo FS, Obiako R, Nichols M, et al. Knowledge and perspectives of community members on risk assessment for stroke prevention using mobile health approaches in Nigeria. J Stroke Cerebrovasc Dis. 2023;32(9):107265.
19. Silvera-Tawil D, Cameron J, Li J, et al. Multicomponent Support Program for Secondary Prevention of Stroke Using Digital Health Technology: Co-Design Study. J Med Internet Res. 2024;26:e54604.
20. Pereira CM, Matos M, Carvalho D, et al. Building Bridges between People with Stroke, Families, and Health Professionals: Development of a Blended Care Program for Self-Management. J Clin Med. 2024;13:300.
21. Paul L, Wyke S, Brewster S, et al. Increasing physical activity in stroke survivors using Starfish, an interactive mobile phone application: a pilot study. Top Stroke Rehabil. 2016;23(3):170–177.
22. Gong E, Gu W, Luo E, et al. Development and Local Contextualization of Mobile Health Messages for Enhancing Disease Management Among Community-Dwelling Stroke Patients in Rural China. JMIR Mhealth Uhealth. 2019;7(12):e15758.
23. Kerr A, Smith M, Reid L, Baillie L. Adoption of Stroke Rehabilitation Technologies by the User Community: Qualitative Study. JMIR Rehabil Assist Technol. 2018;5(2):e15.
24. Requena M, Montiel E, Baladas M, et al. Farmalarm: Application for mobile devices improves risk factor control after stroke. Stroke. 2019;50(7):1819–1824.
25. Zhou B, Zhang J, Zhao Y, et al. Caregiver-delivered stroke rehabilitation in rural China: the RECOVER Randomized Controlled Trial. Stroke. 2019;50:1825–1830.
26. Vloothuis J, de Bruin J, Mulder M, et al. Description of the CARE4STROKE programme: a caregiver-mediated exercises intervention with e-health support. Physiother Res Int. 2018;23:e1719.
27. Thompson AN, Dawson DR, Legasto-Mulvale JM, et al. Mobile Technology-Based Interventions for Stroke Self-Management Support: Scoping Review. JMIR Mhealth Uhealth. 2023;11:e46558.
28. Deutschbein J, Grittner U, Schneider A, Schenk L. Community care coordination for stroke survivors: results of a complex intervention study. BMC Health Serv Res. 2020;20(1).
29. Cruickshank A, Brooks ED, Sperling C, et al. Exploring the experiences of adults with stroke in virtual community-based stroke programs: a qualitative descriptive study. BMC Health Serv Res. 2024;24:600.
30. Reszel J, van den Hoek J, Nguyen T, et al. How Community-Based Teams Use the Stroke Recovery in Motion Implementation Planner: Longitudinal Qualitative Field Test Study. JMIR Form Res. 2022;6(7):e37243.
31. Blanton S, Dunbar S, Clark PC. Content validity and satisfaction with a caregiver-integrated web-based rehabilitation intervention for persons with stroke. Top Stroke Rehabil. 2018;25:168–173.
32. Caunca MR, Simonetto M, Hartley G, et al. Design and usability testing of the stroke caregiver support system: a mobile-friendly website to reduce caregiver burden. Rehabil Nurs. 2018;45:166–177.
33. Sureshkumar K, Murthy G, Natarajan S, et al. Evaluation of the feasibility and acceptability of the 'Care for Stroke' smartphone-enabled intervention. BMJ Open. 2016;6:e009243.
34. Demir Y, Gozum S. Evaluation of Quality, Content, and Use of the Web Site Prepared for Family Members Giving Care to Stroke Patients. Comput Inform Nurs. 2015;33(9):396–403.
35. Favilla CG, Reehal N, Cummings SR, et al. Personalized Video-Based Educational Platform to Improve Stroke Knowledge: A Randomized Clinical Trial. J Am Heart Assoc. 2024;13(15):e035176.
36. Leonardi M, Fheodoroff K. Goal Setting with ICF and Multidisciplinary Team Approach in Stroke Rehabilitation. In: Clinical Pathways in Stroke Rehabilitation. Springer; 2021:35–56.
37. Freund M, Carey M, Dilworth S, et al. Effectiveness of ICT interventions for stroke survivors and their support people: a systematic review. Disabil Rehabil. 2022;44(17):4563–4578.
38. Scrivener K, Sewastenko J, Bouvier-Farrell A, et al. Feasibility of a self-managed, video-guided exercise program for stroke survivors. Stroke Res Treat. 2021;2021:5598100.
39. Smith CE, Levonian Z, Ma H, et al. "I Cannot Do All of This Alone": Exploring support in online health communities. ACM Trans Comput Hum Interact. 2020;27(5):1–41.
40. De Simoni A, Shanks A, Balasooriya-Smeekens C, Mant J. Stroke survivors and families receive support from online forums: descriptive and qualitative study. BMJ Open. 2016;6(4):e010501.
41. Thomas K, Gamlin C, De Simoni A, Mullis R, Mant J. How is poststroke fatigue understood by stroke survivors and carers? A thematic analysis of an online discussion forum. BMJ Open. 2019;9(7):e028958.
42. Smith FE, Jones C, Gracey F, Mullis R, Coulson NS, De Simoni A. Emotional adjustment post-stroke: A qualitative study of an online stroke community. Neuropsychol Rehabil. 2021;31(3):414–431.
43. Lo SHS, Chau JPC, Lau AYL, et al. Virtual Multidisciplinary Stroke Care Clinic for Community-Dwelling Stroke Survivors: A Randomized Controlled Trial. Stroke. 2023;54(10):2482–2490.
44. Saywell NL, Vandal AC, Mudge S, et al. Telerehabilitation After Stroke Using Readily Available Technology: A Randomized Controlled Trial. Neurorehabil Neural Repair. 2021;35(1):88–97.
45. Mainali S, Stutzman S, Sengupta S, et al. Feasibility and efficacy of Nurse-Driven Acute Stroke Care. J Stroke Cerebrovasc Dis. 2017;26:987–991.
46. Lam SKY, Chau JPC, Lo SHS, et al. Evaluation of Cost-Effectiveness of a Virtual Multidisciplinary Stroke Care Clinic. J Am Heart Assoc. 2024;13(17):e035367.
47. Newland P, Sargent R, Van Aman MN, et al. Use of Video Education with Hospitalized Acute Stroke Patients: A Literature Review. MEDSURG Nursing. 2023;32:106.
48. Givon N, Zeilig G, Weingarden HRD. Video-games used in a group setting to improve physical activity in chronic stroke: a randomized controlled trial. Clin Rehabil. 2016;30(4):383–392.
49. Tsang WN, Lee JJ, Yang SC, et al. Stroke caregivers' perception on instant messaging application use for psychological intervention. Psychol Health Med. 2024;29(7):1208–1221.
50. Johnson L, Bird ML, Muthalib M, Teo WP. STRIVE Online Platform for Stroke Survivors: A Randomized Controlled Trial. Arch Phys Med Rehabil. 2020;101(7):1131–1137.
51. Lo SHS, Chau JPC, Choi KC, et al. Stroke survivor and caregiver experiences of virtual reality gaming to promote social participation: A qualitative study. PLoS ONE. 2024;19(12):e0315826.
52. Lee M, Son J, Kim J, et al. Comparison of individualized virtual reality- and group-based rehabilitation in older adults with chronic stroke. Eur J Integr Med. 2016;8(5):738–746.
53. Krishnan S, Mandala MA, Wolf SL, et al. Perceptions of stroke survivors regarding adoption of technology and exergames. PM R. 2023;15(11):1403–1410.
54. Luo Z, Lim AE, Durairaj P, et al. Development of a compensation-aware virtual rehabilitation system for upper extremity rehabilitation. J Neuroeng Rehabil. 2023;20(1):56.
55. Sun X, Ding J, Dong Y, Ma X, et al. A Survey of Technologies Facilitating Home and Community-Based Stroke Rehabilitation. Int J Hum Comput Interact. 2022;39(5):1016–1042.
56. Giachero A, Calati M, Pia L, et al. Conversational therapy through semi-immersive virtual reality environments for post-stroke aphasia. Behav Neurol. 2020;2020:2846046.
57. Peters DM, O'Brien ES, Kamrud KE, et al. Utilization of wearable technology to assess gait and mobility post-stroke: a systematic review. J Neuroeng Rehabil. 2021;18(1):67.
58. Kamwesiga JT, Tham K, Guidetti S. Experiences of using mobile phones among persons with stroke and their families in Uganda. Disabil Rehabil. 2017;39(5):438–449.
59. Lee SI, Adans-Dester CP, Grimaldi M, et al. Wearable sensor-based approach for upper-limb motor training in stroke rehabilitation. IEEE J Transl Eng Health Med. 2018;6:1–11.
60. Demers M, Bishop L, Cain A, et al. Wearable Technology to Capture Arm Use of People With Stroke in Home and Community Settings. Phys Ther. 2024;104(2):pzad172.
